# A miR-125b/CSF1-CX3CL1/tumor-associated macrophage recruitment axis controls testicular germ cell tumor growth

**DOI:** 10.1038/s41419-018-1021-z

**Published:** 2018-09-20

**Authors:** Aalia Batool, Yu-Qian Wang, Xiao-Xia Hao, Su-Ren Chen, Yi-Xun Liu

**Affiliations:** 1 State Key Laboratory of Stem Cell and Reproductive Biology, Institute of Zoology, Chinese Academy of Sciences, 100101 Beijing, China; 2 University of Chinese Academy of Sciences, 100049 Beijing, China

## Abstract

Tumor growth is modulated by crosstalk between cancer cells and the tumor microenvironment. Recent advances have shown that miRNA dysfunction in tumor cells can modulate the tumor microenvironment to indirectly determine their progression. However, this process is poorly understood in testicular germ cell tumors (TGCTs). We reported here that miR-125b was repressed in TGCT samples by epigenetic modifications rather than genetic alternations. Furthermore, miR-125b overexpression significantly alleviated the tumor growth in two NCCIT human embryonic carcinoma xenograft models in vivo, whereas miR-125b did not stimulate autonomous tumor cell growth in vitro. Notably, forced expression of miR-125b in NCCIT embryonic carcinoma cells decreased the abundance of host tumor-associated macrophages (TAMs) within tumor microenvironment. Selective deletion of host macrophages by clodronate abolished the anti-tumoral ability of miR-125b in xenograft models. By RNA profiling, Western blot and luciferase reporter assay, we further observed that miR-125b directly regulated tumor cell-derived chemokine CSF1 and CX3CL1, which are known to control the recruitment of TAMs to tumor sites. Lastly, we found that one set of miRNAs, which are under the regulation of miR-125b, might convergently target CSF1/CX3CL1 in NCCIT cells using miRNA profiling. These findings uncover the anticancer effect of miR-125b via mediating tumor-stroma crosstalk in xenograft models of TGCTs and raise the possibility of targeting miR-125b as miRNA therapeutics.

## Introduction

Testicular germ cell tumors (TGCTs) are one of the most frequent solid tumors of adolescents and young adult males, which approximately account for 8.9% of tumors among 20–39 year-old males worldwide in 2012^1,2^. Histologically, TGCTs can be divided into seminoma and non-seminoma (including embryonic carcinoma, teratoma, and yolk sac)^3^. Seminoma is highly similar to primordial germ cells, while embryonic carcinoma is malignant counterparts of embryonic stem cells^4^. According to the European Association of Urology testis cancer guidelines, approximately 15–20% of stage I seminoma patients and up to 30% of stage I nonseminoma patients have subclinical metastatic disease and will relapse after orchiectomy^5,6^. Although the cure rate of TGCTs is relatively high, exploration of mechanisms underlying the occurrence, progression, recurrence and chemotherapeutic sensitivity^7^ and clinical therapeutics without long-term side effects^6^ are needed to reduce the cancer burden in this underserved age group.

Most cancer research has focused upon intrinsic properties of tumor cells (e.g., proliferation, apoptosis) and corresponding therapeutics are directed against these tumor cells. However, targeting of tumor cells is not equivalent to targeting of tumor tissues. Recently, advances in cancer research have emphasized that tumor cells display extensive and dynamic cross-talk with the neoplastic microenvironment^8–11^. Tumor microenvironment is highly heterogeneous, mainly containing lymphocytes (e.g., T cells, B cells, and natural killer cells), endothelial cells, tumor-associated macrophages (TAMs), cancer-associated fibroblasts, myeloid-derived suppressor cells, local and bone marrow-derived stem/progenitor cells, and surrounding stroma^12^. Although the tumor growth-promoting ability of TAMs has been extensively studied^13,14^, it is still not clear whether TAMs are reciprocally controlled by developmental programs that are activated in tumor cells.

Can microRNAs (miRNAs) drive the communication between tumor cells and tumor microenvironment? Recent advances support this hypothesis, showing that miRNA dysfunction in tumor cells can modulate various aspects of tumor microenvironment, including angiogenesis^15^, immune cell recruitment^16^, extracellular matrix remodeling^17^, immunosuppression^18^, and metastasis^19^. miRNAs are short non-coding RNAs that modulate gene expression post-transcriptionally, either by inhibiting translation or by causing degradation through binding to the 3’ untranslated (UTR) regions of target messenger RNAs^20^. In addition to physiological conditions, miRNAs are deeply involved in tumor onset and progression, either behaving as oncomiRNAs or as tumor suppressor miRNAs^21^. However, remarkably little is known about miRNA regulation of the communication between tumor cells and TAMs, a predominant component of tumor microenvironment.

miR-125b functions as a tumor suppressor miRNA in a variety of tumors through regulating intrinsic properties of tumor cells, including proliferation, apoptosis, and stem-like characteristics^22–25^. Here we report that the miR-125b can act through a different mechanism to control TGCT growth, as low miR-125b expression in tumor cells promotes a TAM-rich microenvironment via increasing the production of tumor-derived chemokine CSF1 and CX3CL1 for TAM recruitment. Our findings support a model in which epigenetically repressed miR-125b in tumor cells creates a permissive microenvironment for the growth of TGCT xenografts.

## Results

### Low miR-125b expression in TGCTs

**Fig. 1:**
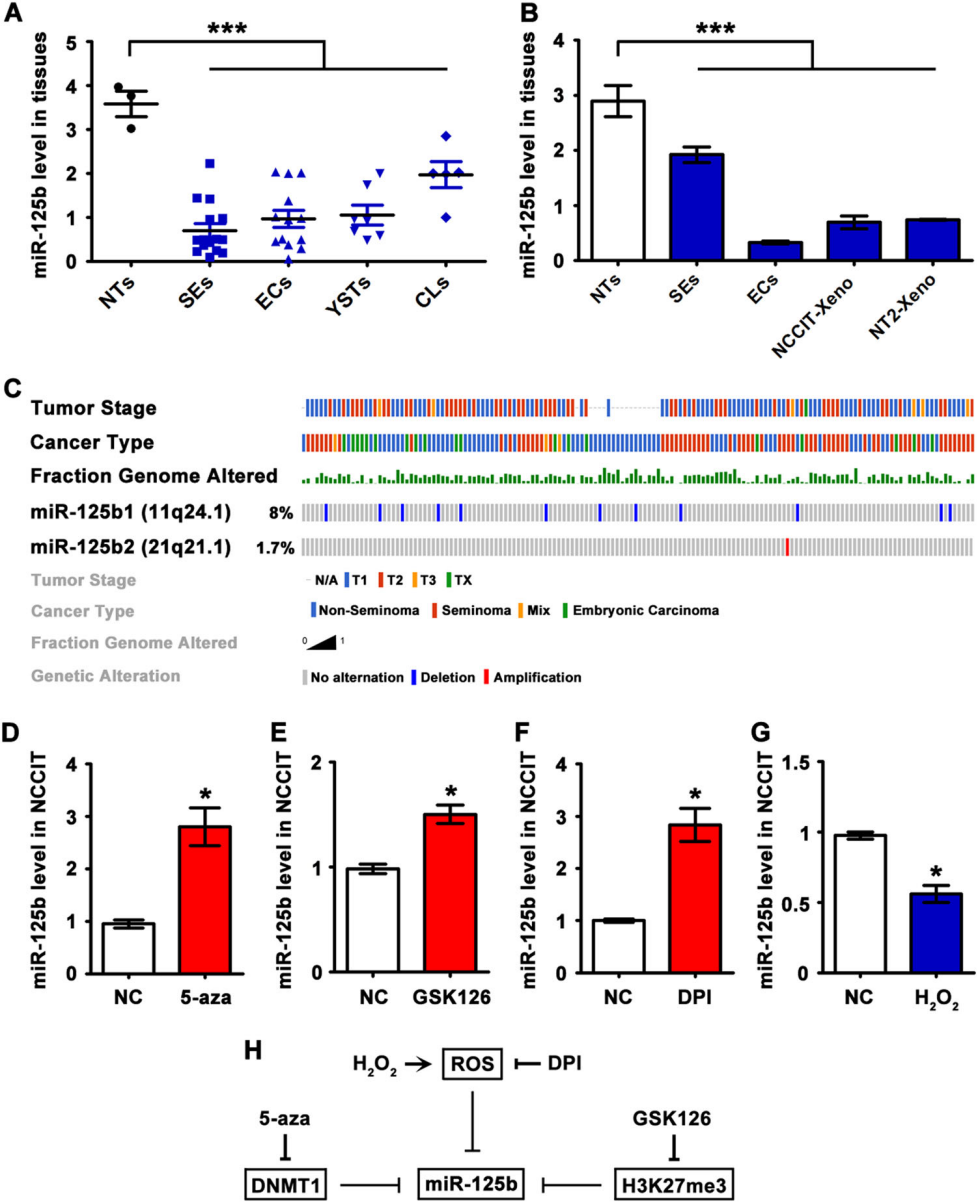
Repressed miR-125b expression in TGCTs by epigenetic modifications. **a** miR-125b level in normal testes (NTs, *n* = 3), seminomas (SEs, *n* = 15), embryonic carcinomas (ECs, *n* = 13), yolk sac tumors (YSTs, *n* = 7), and EC-derived cell lines (CLs, *n* = 5); re-analyzed data from Gillis et al^26^. **b** Relative level of miR-125b in NTs, SEs, ECs, NCCIT-xenografts, and NT2-xenografts by qRT-PCR (*n* = 3 each). Asterisks(***) indicated *p* < 0.0001 with a one-way ANOVA. Data were presented as the mean ± SEM. **c** Copy-number status of precursor miR-125b (miR-125b1 and miR-125b2) in 156 TGCT patients in the TCGA CNV array dataset. Tumor stage, cancer type, and fraction genome alternation of individual sample were indicated. **d**–**g** qRT-PCR analysis (*n* = 3 each) of miR-125b level in NCCIT cells treated with 5-aza (5 µM for 72 h) (**d**), or GSK126 (1000 nM for 72 h) (**e**), or DPI (1.5 µM for 12 h) (**f**) or H_2_O_2_ (25 µM for 4 h) (**g**). U6 was served as endogenous control for normalization. Asterisks(*) indicated *p* < 0.05 by student’s *t* test. Data were presented as the mean ± SEM. **h** Schematic diagram showing that miR-125b was repressed via epigenetic modifications in TGCTs

For comparison of miR-125b expression between TGCTs and normal testes, we extracted and re-analyzed global TaqMan miRNA profiling data from the study of Gillis et al^26^. We found that miR-125b level was relatively low in seminomas (SEs, *n* = 15), embryonic carcinomas (ECs, *n* = 13), yolk sac tumors (YSTs, *n* = 7), and EC-derived cell lines (CLs, *n* = 5) compared to normal testes (NTs, *n* = 3) (Fig. 1a). To validate this observation, we further examined the miR-125b level in normal adult testicular tissues, TGCT tissues (seminoma and embryonic carcinoma), and tumor cell line (NCCIT and NT2) xenografts using qRT-PCR. As expected, miR-125b exhibited significantly low expression in both TGCT tissues and tumor cell line xenografts than in normal adult testicular samples, inferring a potential role of miR-125b repression in TGCT progression (Fig. 1b).

### Mechanisms involved in miR-125b repression in TGCTs

Genetic alternations and epigenetic regulations are two major mechanisms controlling the spatial and temporal expression of miRNAs^27^. Analysis of high-resolution copy-number variation (CNV) datasets from The Cancer Genome Atlas (TCGA) demonstrated that deletions (approximately 8%) and amplifications (approximately 1.7%) of precursor miR-125b (miR-125b1 and miR-125b2) were barely detected in 156 TGCT samples (Fig. 1c), indicating that repressed miR-125b expression in TGCTs was unlikely due to genetic alternations. To validate the possibility of epigenetic regulations, we treated human embryonic carcinoma NCCIT cells with different chemical drugs: 5-aza (DNA methyltransferase inhibitor^28^), GSK126 (eliminator of H3K27me3 histone modification^29^), DPI (NADPH oxidase-dependent reactive oxygen species (ROS) inhibitor^30^) and H_2_O_2_ (a major source of ROS^30^), respectively. Notably, treatment of 5-aza, GSK126 and DPI significantly increased the expression of miR-125b in NCCIT cells compared with untreated cells (Fig. 1d–f). After H_2_O_2_ treatment, miR-125b expression was markedly reduced by approximately 30% (Fig. 1g). These findings indicated that miR-125b in embryonic carcinoma was mainly repressed by epigenetic modifications, including DNA methylation, histone modifications and ROS (Fig. 1h).

### miR-125b inhibits TGCT growth in vivo but not in vitro

**Fig. 2:**
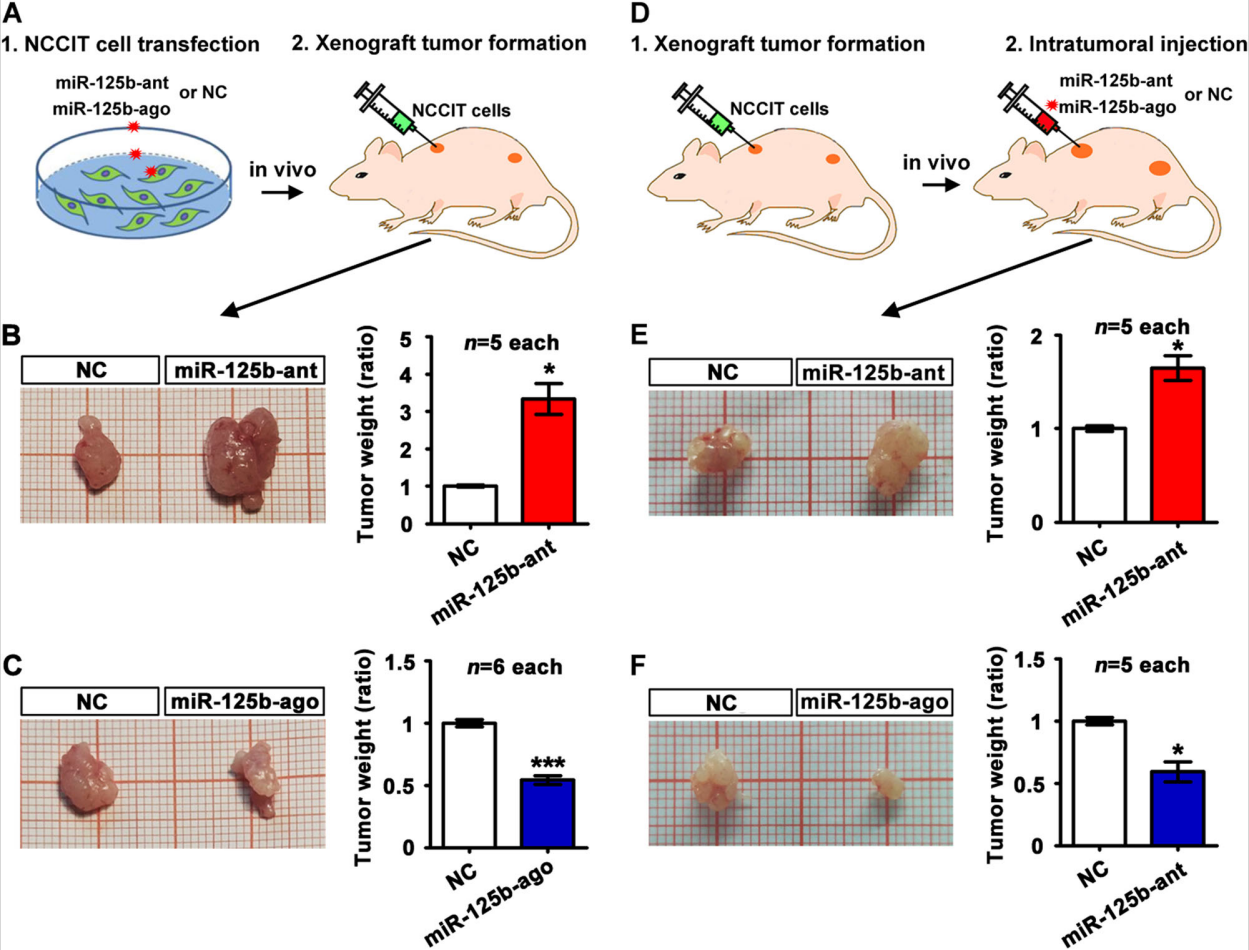
Anti-tumoral effect of miR-125b on TGCT growth in vivo. **a** Experimental procedure for subcutaneous transplantation of miR-125b antagomir-targeting, or agomir, or their non-targeting controls (NC)-transfected NCCIT cells into nude mice. **b** Relative tumor weight of xenografts derived from NC and miR-125b antagomir-transfected NCCIT cells (*n* = 5 each). **c** Reduced tumor weight in nude mice injected with miR-125 agomir-transfected NCCIT cells, compared with tumor weight in NC groups (*n* = 6 each). **d** Experimental procedure for intratumoral injection of oligonucleotides. Procedural details were provided in Materials and Methods. **e** Intratumoral injection of miR-125b antagomir accelerated the growth of already-formed xenografts (*n* = 5 each). **f** Relative tumor weight of already-formed xenografts after intratumoral injection of miR-125b agomir or NCs (*n* = 6 each). Asterisks(*) indicated *p* < 0.05 and *** indicated *p* < 0.0001 by student’s *t* test. Data were presented as the mean ± SEM

To investigate the effect of miR-125b on tumor growth in vivo, NCCIT cells transfected with miR-125b antagomir, or miR-125b agomir, or their negative controls were subcutaneously transplanted into the flanks of nude mice (Fig. 2a). qRT-PCR analysis revealed that miR-125b expression decreased by 70% in NCCIT cells treated with miR-125b antagomir compared to the negative control treatment. Conversely, miR-125 abundance was significantly increased (approximately thousand-fold) in NCCIT cells after miR-125b agomir transfection (Fig. S1). Xenograft tumor was significantly larger in miR-125b antagomir group, compared to the negative control (Fig. 2b), while miR-125b agomir pretreatment markedly decreased xenograft tumor growth (Fig. 2c). To mimic the clinical situation of tumor therapies, we further adopt the intratumoral injection approach in tumor-bearing mice (Fig. 2d). Briefly, miR-125b antagomir, or miR-125b agomir, or their negative controls was injected into the tumor xenografts of similar size. We found that the weight of miR-125b antagomir-injected NCCIT xenografts was approximately 50% heavier than that of the negative control group (Fig. 2e). Conversely, intratumoral injection of miR-125b agomir significantly reduced xenograft tumor weight (Fig. 2f). Collectively, we confirmed the suppressive effect of miR-125b on NCCIT tumor growth in vivo using two xenograft models.

**Fig. 3:**
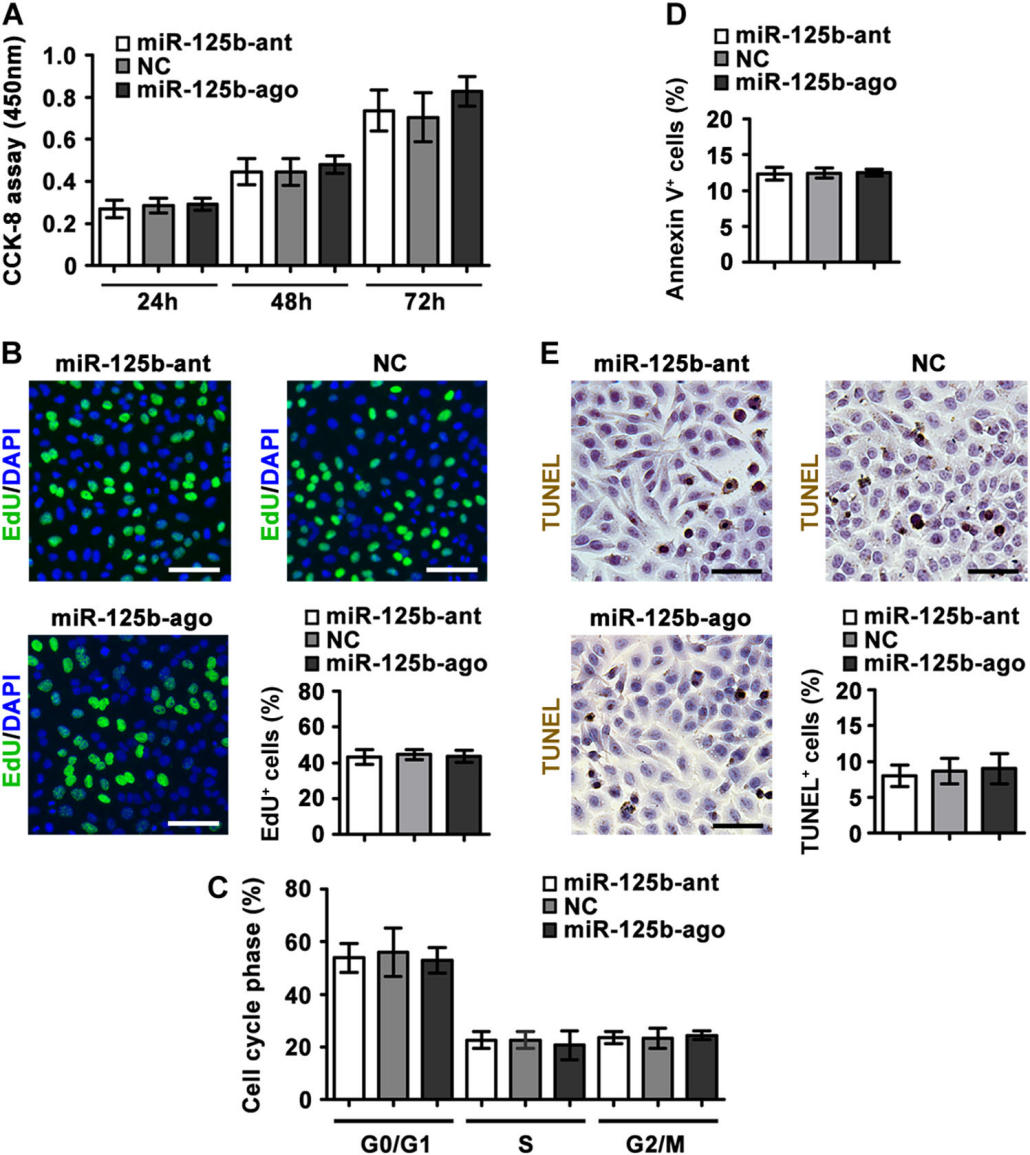
No effect of miR-125b on NCCIT cells in vitro. **a** CCK-8 assay of NCCIT cells at the indicated time points (24 h, 48 h, and 72 h) after transfection of miR-125b antagomir, or agomir, or their NCs. **b** Representative EdU incorporation staining and the percentage of EdU-positive cells were shown. **c** Cell cycle assay by flow cytometric analysis of PI-stained nuclei. **d** Apoptosis assay of transfected NCCIT cells by flow cytometric analysis of Annexin V-FITC/PI staining. **e** Representative TUNEL staining and quantification of fraction of TUNEL-positive cells were shown. Data were presented as the mean ± SEM (*n* = 3, each group). Scale bar in **b** and E, 25 μm

Surprisingly, proliferation, cell cycle and apoptosis of miR-125b-knockdown, -overexpression, and control NCCIT cells were identical in vitro, as revealed by CCK-8 assay and EdU incorporation staining (Fig. 3a, b), flow cytometric analysis of cell cycle phase (Fig. 3c), flow cytometric analysis of Annexin V-FITC/PI and TUNEL staining (Fig. 3d, e), respectively. The different phenotypes obtained from in vivo and in vitro suggest that the ability of miR-125b to limit tumor growth in vivo may depend on interactions with host cells.

### Tumor cell miR-125b regulates host macrophage recruitment

**Fig. 4:**
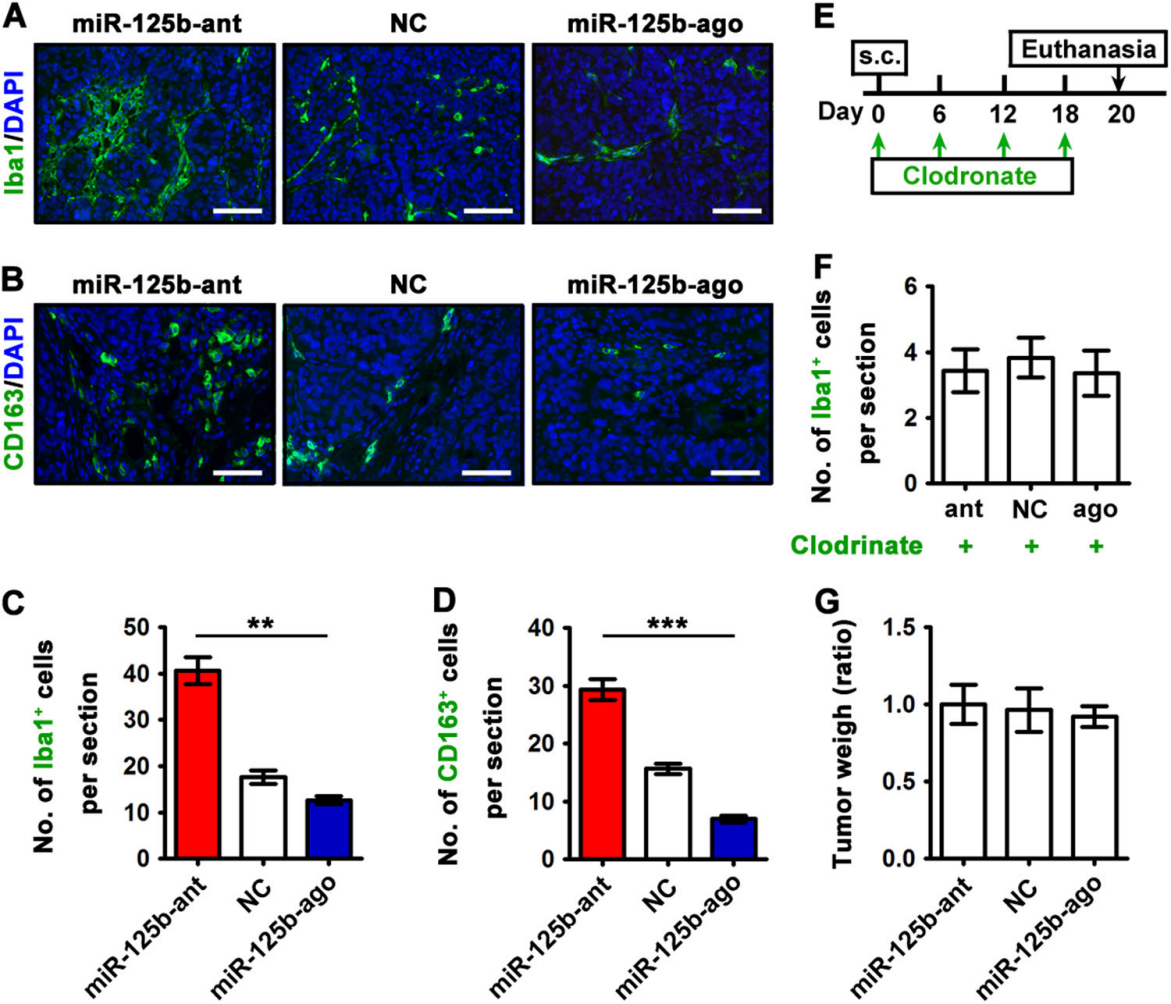
miR-125b in tumor cells controls the abundance of host macrophages in tumor microenvironment. **a, b** Immunofluorescence staining for macrophage markers of Iba1 (**a**) and CD163 (**b**) on the tissue sections of miR-125b antagomir-, or agomir-, or NC-transfected NCCIT xenografts was shown. Scale bar, 100 μm. **c, d** The density of Iba1-positive (**c**) or CD163-positive (**d**) macrophages per section of miR-125b antagomir-, or agomir-, or NC-transfected NCCIT xenografts. **e** Transfected NCCIT cells were subcutaneously (s.c.) xenografted into the flank of nude mice on day 0. Clodronate liposomes were administered every six days for three weeks (i.e., on days 0, 6, 12, and 18). **f** Average number of Iba1-positve macrophages per section after successive clodronate liposome treatment. **g** Relative tumor weight of miR-125b antagomir-trasfected, agomir-transfected, and NC-transfected NCCIT xenografts in nude mice received clodronate treatment (*n* = 3 each). Data were presented as the mean ± SEM. Asterisks(**) indicated *p* < 0.01 and *** indicated *p* < 0.0001 by one-way ANOVA

Taken tumor microenvironment into account, we hypothesized that low miR-125b-expressed tumor cells could promote a permissive microenvironment for tumor growth. To test this hypothesis, we performed immunofluorescence staining of xenograft sections. Notably, reduced number of Iba1- and CD163-positive tumor-associated macrophages (TAMs) was observed in xenografts derived from miR-125 agomir-treated tumor cells. On the contrary, miR-125 antagomir-treated tumors recruited more TAMs (Fig. 4a-d). To determine whether host TAMs account for miR-125b level-dependent NCCIT xenograft growth, we utilized clodronate-containing liposomes to selectively deplete macrophages in nude mice (Fig. 4e, f). Notably, the anti-tumoral role of miR-125b was largely eliminated by deletion of host macrophages, as indicated by the similar tumor size among miR-125b-knockdown, -overexpression, and control xenografts (Fig. 4g). Collectively, these results suggest that miR-125b exerts anti-tumoral function mainly via tumor-induced mobilization of pro-tumorigenic host TAMs into tumor microenvironment.

### miR-125b targets chemokines responsible for the recruitment of host TAMs

**Fig. 5:**
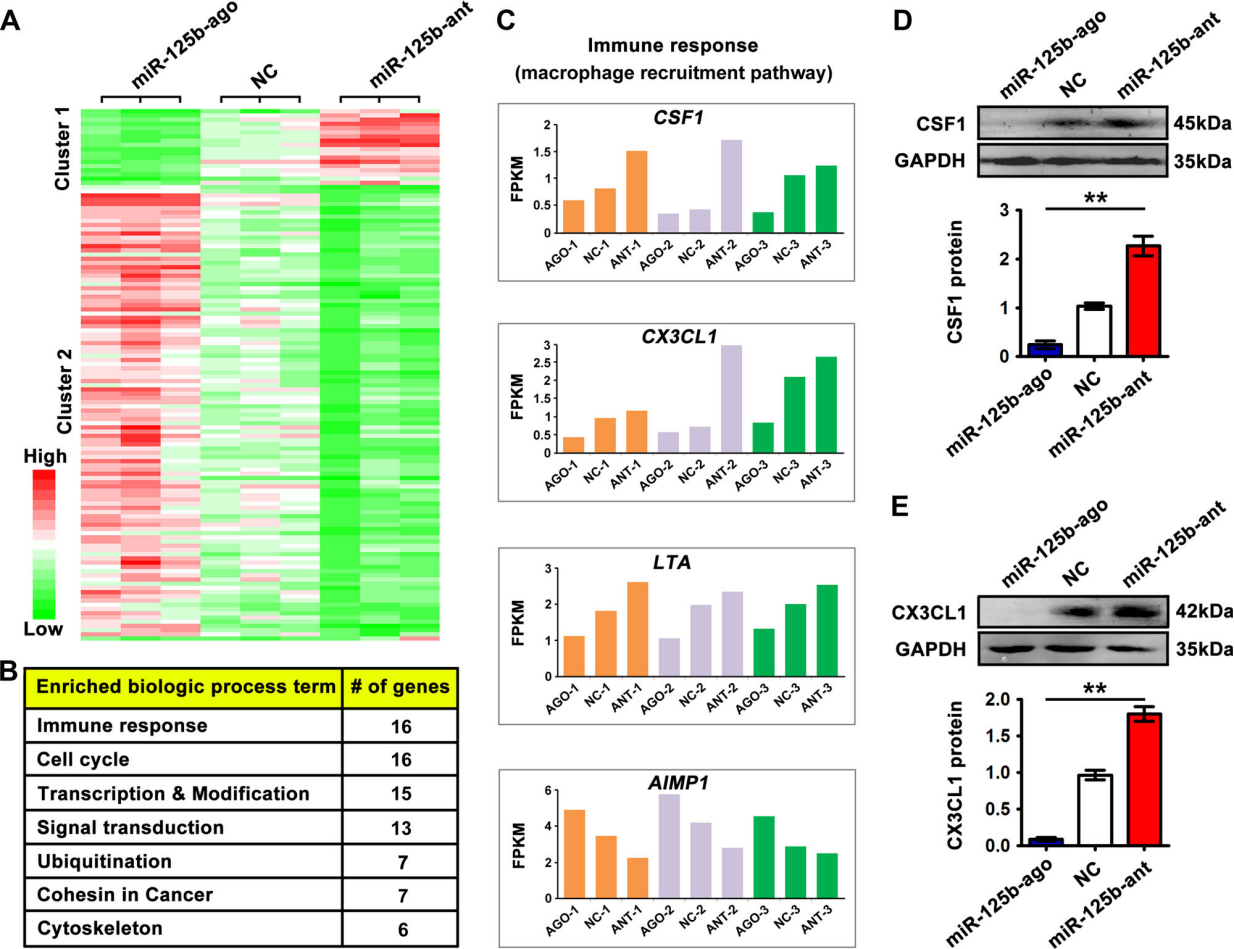
Transcriptome analysis reveals gene regulation under miR-125b in NCCIT tumor cells. **a** Heatmap displaying 125 differentially expressed genes among miR-125b antagomir-, miR-125b agomir-, and NC-transfected NCCIT cells (Table S1). Differentially expressed genes were further clustered into two distinct clusters (cluster 1: miR-125b-negatively regulated genes, *n* = 18; cluster 2: miR-125b-positively regulated genes, *n* = 107). **b** Table of enriched biologic processes by KEGG analysis of differentially expressed genes. **c** Expression levels (FPKM values) of *CSF1*, *CX3CL1*, *LTA,* and *AIMP1* that are suggested to exert macrophage recruitment activities. **d, e** Protein levels of CSF1 (**d**) and CX3CL1 (**e**) were detected in miR-125b knockdown, miR-125b overexpression, and NC NCCIT cells by Western blot analysis and normalized to GAPDH. Asterisks(**) indicates *p* < 0.01 by one-way ANOVA. Data were presented as the mean ± SEM

To investigate the mechanism underlying the inhibitory effect of miR-125b on TAM density and NCCIT xenograft growth without bias, we analyzed differentially expressed genes among miR-125b antagomir-transfected, miR-125b agomir-transfected, and negative control-transfected NCCIT tumor cells by RNA sequencing. The expression levels of 18 genes (cluster 1) were increased in miR-125b antagomir-transfected cells; meanwhile, they were downregulated in miR-125b agomir-treated group. Of these, *BCL-3* has been previously shown to be the target of miR-125b in ovary cancer^31,32^. A second class of 107 genes was positively regulated by miR-125b (Fig. 5a, Table S1). Cluster analysis revealed that the top seven enriched pathways, in sequences, were immune response, cell cycle, transcription and modification, signal transduction, ubiquitination, cohesin in cancer, and cytoskeleton (Fig. 5b, Table S2). Notably, expression of genes implicated in macrophage recruitment pathway, such as *CSF1*, *CX3CL1*, *LTA,* and *AIMP1*, was negatively regulated by miR-125b in NCCIT cells (Fig. 5c). Furthermore, western blot analysis revealed that the protein levels of CSF1 and CX3CL1 were significantly reduced in miR-125-overexpressed cells, while their levels were increased in miR-125b-silenced cells (Fig. 5d, e). In agreement with transcriptome and western blot data, we found that the concentrations of CSF1 and CX3CL1 in both cell lysates and cell culture supernatants were significantly elevated in miR-125b antagomir-treated groups, while their levels were relative low in miR-125b agomir-treated groups compared to controls (Fig. S3). Collectively, miR-125b abundance in tumor cells determines TAM recruitment at least partly by regulating chemokine CSF1 and CX3CL1 directly and/or indirectly.

### CSF1 and CX3CL1 are direct targets of miR-125b in NCCIT cells

**Fig. 6:**
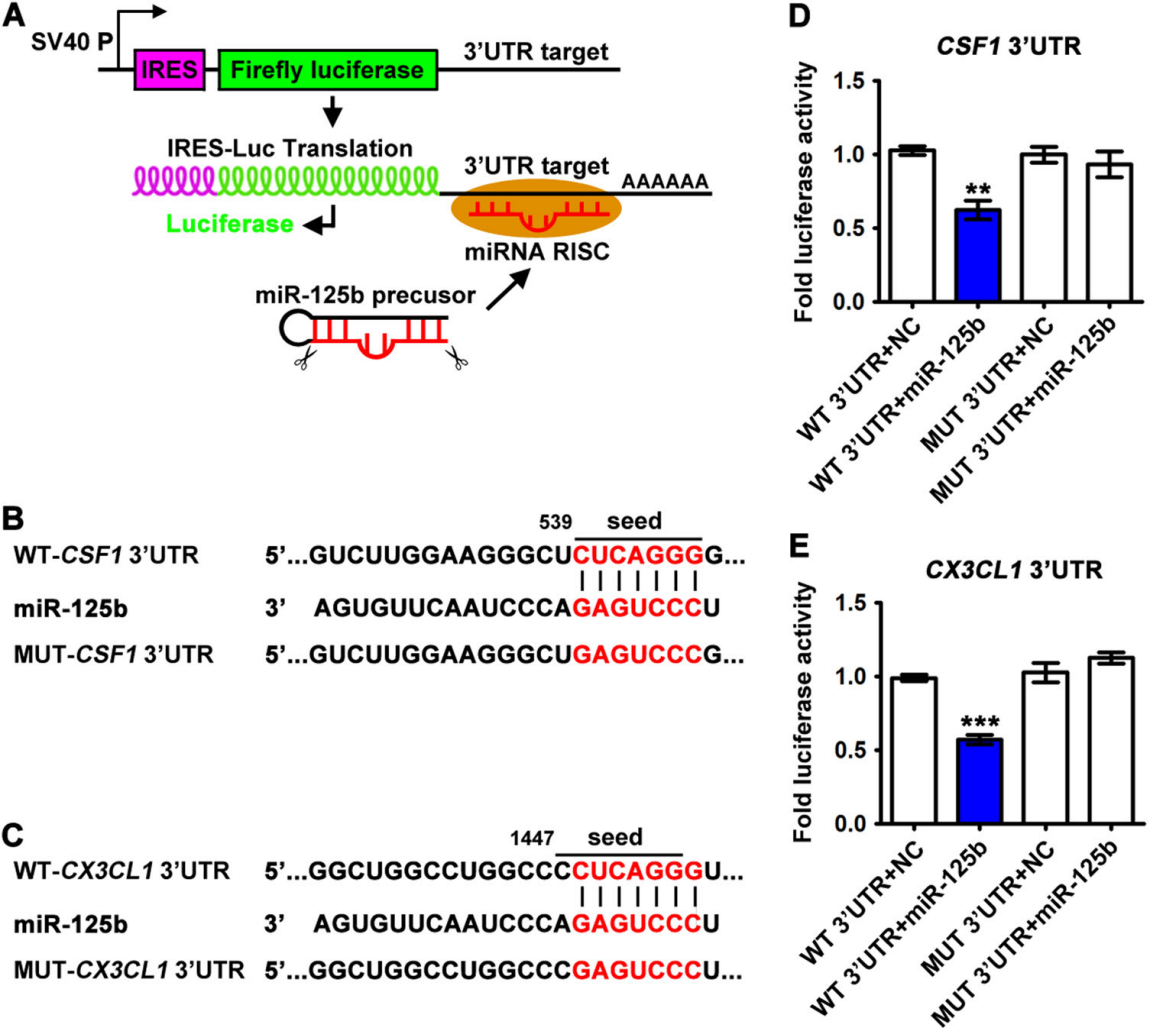
*CSF1* and *CX3CL1* are served as direct targets of miR-125b in NCCIT tumor cells. **a** A schematic representation of the 3’ UTR luciferase reporter system and pMirTarget plasmid was shown. **b, c** 3’ UTRs of *CSF1* (**b**) and *CX3CL1* (**c**) harbor conserved seed match sequences (7-mer, CUCAGGG) for miR-125b recognition. **d, e** Luciferase assay in NCCIT cells was performed by co-transfection of Renilla, reporter plasmids carrying wild-type (WT) or mutant (MUT) 3’UTR, and miR-125b mimic or NC. Fold luciferase activity was displayed by the Firefly/Renilla ratio. Asterisks(**) indicated *p* < 0.01 and *** indicated *p* < 0.0001 by one-way ANOVA. Data were presented as the mean ± SEM

To validate CSF1 and CX3CL1 are post-transcriptionally regulated by miR-125b, 3’ untranslated region (UTR) of *CSF1* (or *CX3CL1*) gene was cloned downstream of the firefly luciferase gene of pMirTarget luciferase reporter plasmids. The chimeric translation level is regulated by its interaction with miRNA-RISC (RNA-induced silencing complex) and is quantified by a colorimetric assay (Fig. 6a). The luciferase reporter plasmid pMirTarget-*CSF1* (or *CX3CL1*)-3’ UTR or mutant reporter plasmid carrying point mutations in the putative miR-125b seeding sites was co-transfected with miR-125b mimic or mimic NC, separately (Fig. 6b, c). Reporter assay revealed that miR-125b mimic decreased the luciferase activity of the wild-type (WT)*-CSF1* 3’ UTR and WT-*CX3CL1* 3’ UTR by approximately 42% and 40%, respectively (Fig. 6d, e; column 1 and 2). However, point mutations of the seeding sequence on the 3’ UTR (MUT-*CSF1* 3’ UTR and MUT-*CX3CL1* 3’ UTR) diminished above effect of miR-125b on luciferase activity (Fig. 6d, e; column 3 and 4). These results suggest that the miR-125b binds directly to the predicted binding site in the *CSF1* and *CX3CL1* 3’ UTR in NCCIT tumor cells.

### miR-125b regulates CSF1 and CX3CL1 likely through a miRNA network

**Fig. 7:**
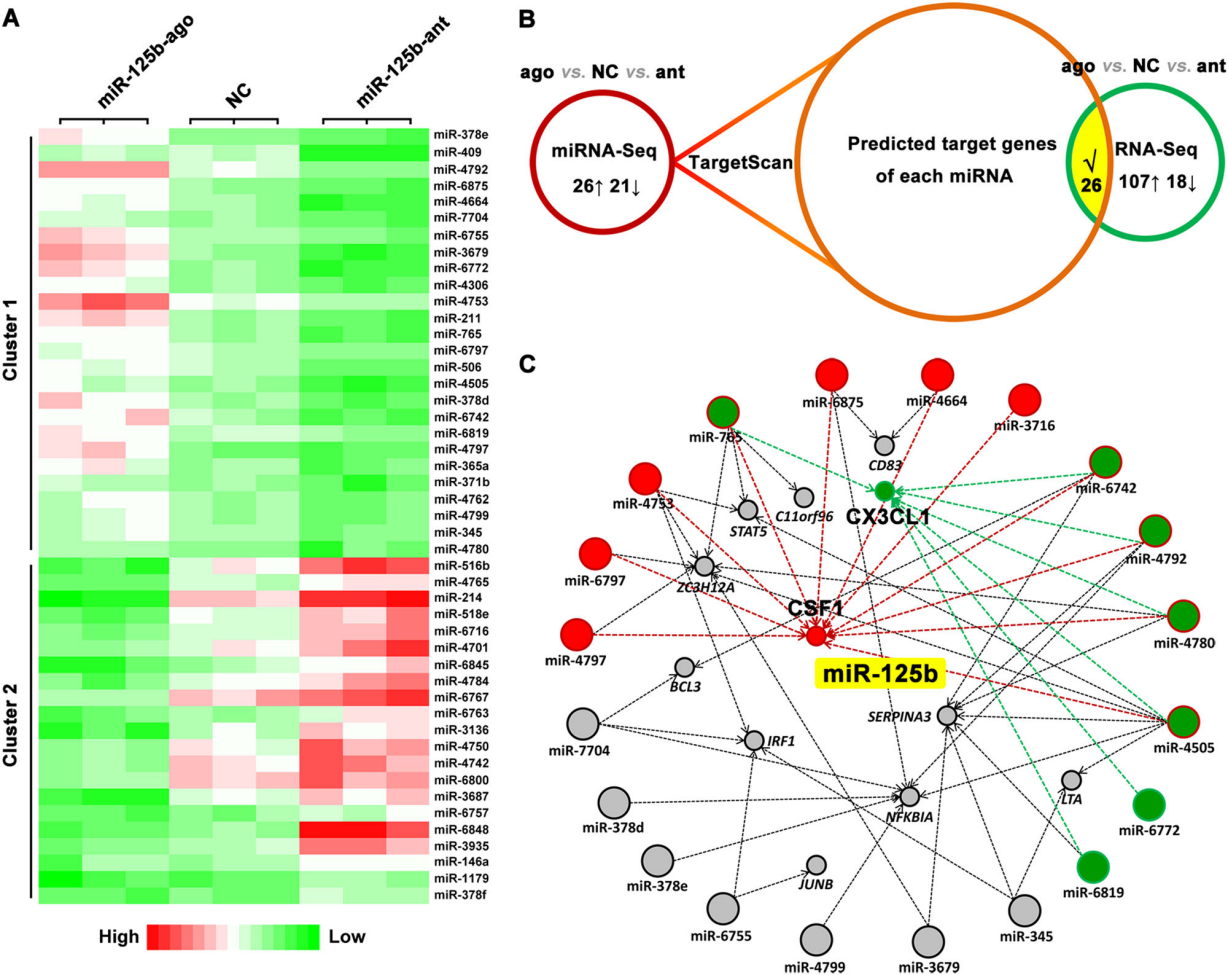
miR-125b regulates *CSF1* and *CX3CL1* likely via a miRNA-mRNA network. **a** Heatmap displaying 47 differentially expressed genes among miR-125b antagomir-, miR-125b agomir-, and NC-transfected NCCIT tumor cells. Differentially expressed genes were further clustered into cluster 1 (genes positively regulated by miR-125b, *n* = 26) and cluster 2 (miR-125b-negatively regulated genes, *n* = 21). **b** Experimental procedure: overlap of predicted targets (by TargetScan) of differentially expressed miRNAs (revealed by miRNA-Seq) and differentially expressed mRNAs (revealed by mRNA-Seq). **c** Interaction network between miRNAs and target mRNAs. Red color indicated interaction association between miRNAs and *CSF1*. Green color highlighted network between miRNAs and *CX3CL1*

To gain further insight into the regulation of CSF1 and CX3CL1 by miR-125b in embryonic carcinoma, we performed miRNA-Seq of miR-125b antagomir-, miR-125b agomir-, and negative control-transfected NCCIT cells. miR-125b exerted positive or negative regulation on 26 and 21 miRNAs, respectively (Fig. 7a). We then screened the overlap of predicted targets of differentially expressed miRNAs (by TargetScan) and differentially expressed mRNAs (from RNA-Seq) (Fig. 7b). Interestingly, a set of miRNAs that are positively regulated by miR-125b, such as miR-4797, miR-6797, miR-4753, miR-765, miR-6975, miR-4664, miR-3716, miR-6742, miR-4792, miR-4780, and miR-4505, combinatorially targeted CSF1 (Fig. 7c). Similarly, miR-765, miR-6742, miR-4792, miR-4780, miR-4505, miR-6772, and miR-6819 were predicted to coordinately regulate CX3CL1 (Fig. 7c). Together, these data suggest that a miRNA network combinationally targeting the core regulons (e.g., CSF1, CX3CL1) may be guaranteed by miR-125b in NCCIT tumor cells.

## Discussion

**Fig. 8:**
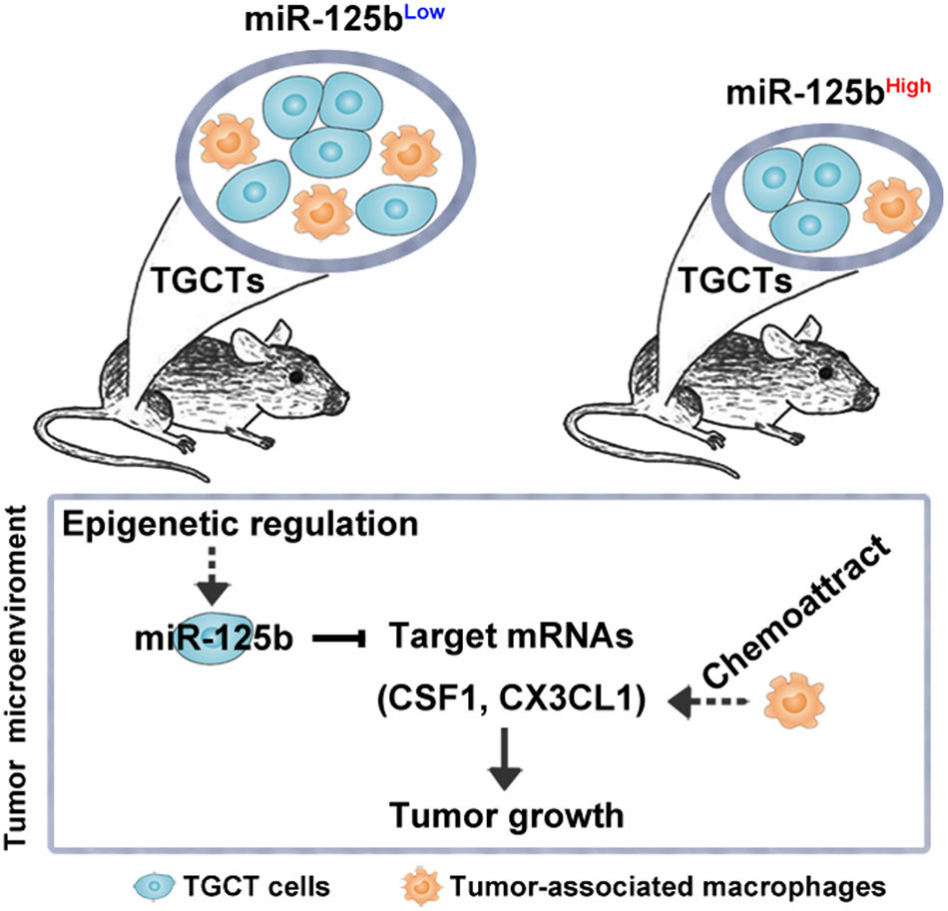
Possible mechanisms of miR-125b-mediated TAM recruitment and TGCT growth in vivo. Epigenetic modifications could account for the repressed miR-125b expression in TGCTs. miR-125b overexpression significantly alleviated the tumor growth in vivo, likely due to the reduced abundance of TAMs. In mechanism, miR-125b directly regulates the expression of tumor cell-derived chemokine CSF1 and CX3CL1, which are known to control the recruitment of TAMs to tumor sites

Here, we show that repressed miR-125b in tumor cells promotes TGCT xenograft growth at least partly through stimulating the recruitment of host tumor growth promotive TAMs (Fig. 8). Our findings in favor of this notion are as follows: (i) the impaired TAM recruitment and NCCIT xenograft tumor growth after the ectopic overexpression of miR-125b in tumor cells, (ii) the abolished effects of miR-125b on NCCIT xenograft tumor growth by the deletion of host macrophages, (iii) the direct regulation of tumor-derived chemokines (e.g., CSF1, CX3CL1) for host TAM recruitment by miR-125b in tumor cells, and (iv) a set of miRNAs under miR-125 regulation combinatorially target CSF1 and CX3CL1 in NCCIT cells.

Our study starts from exploring mechanisms of repressed miR-125b in TGCT samples. Apart from the genetic alterations, epigenetics modifications such as DNA methylation and histone modifications around promoter regions can modify chromatin structure and regulate expression of genes and miRNAs. miR-125b exhibits repressed expression and tumor suppressor activity in many types of cancer^33^ and promoter hypermethylation associated with repressed expression has been shown for miR-125b in breast cancer^25,34^, ovary cancer^30^, and colorectal cancer^35^. Furthermore, H3K9me3 and H3K27me3 histone modifications of miR-125b promoter is demonstrated in breast cancer cell lines^29^. Biological events such as reactive oxygen species (ROS) can also inhibit miRNA expression through increasing the promoter methylation^30^. Taking advantage of inhibitory experiments, we summarize that repression of miR-125b in NCCIT tumor cells is under the combined action of DNA methylation, histone modifications and ROS (Fig. 1), but a mechanism for initiation of this depression has not yet been identified. Based on expression studies, we further demonstrate the significance of repressed miR-125b in TGCT growth using NCCIT xenograft models.

Our observation of negative regulation of TAM recruitment by miR-125b agrees with the established notion that TAMs can stimulate tumor growth and a high frequency of infiltrating TAMs correlates with poor disease outcome^13,36–38^. TAMs derive from circulating monocytic precursors from bone marrow and spleen and infiltrate most solid tumors^39–41^. The recruitment of TAMs to the tumor microenvironment and the differentiation of TAMs toward a pro-tumorigenic phenotype are precisely regulated by tumor cells that secret a wide range of chemokines and cytokines. It is well-demonstrated that CSF1 and CX3CL1 control the recruitment of TAMs into tumor microenvironment. *CSF1* silencing in tumor cells reduces host macrophage invasion, angiogenic activity, and tumor xenograft growth in mice^42,43^. Similarly, *CX3CR1* deficiency in the environment significantly suppresses macrophage accumulation, tumor growth and metastasis^44,45^. Accordingly, targeting TAMs via the CSF1/CSF1R or CX3CL1/CX3CR1 signaling pathway is an attractive therapy for cancers associated with increased numbers of TAMs (reviewed in refs.^46,47^). Similar to other solid tumors, both human TGCT tissues and experimental animal xenografts are infiltrated by TAMs and selective deletion of macrophages in recipient mice by clodronate liposomes abolishes this infiltration and limits tumor growth (Fig. 4). Our study suggests that miR-125b in tumor cells regulates the production of CSF1 and CX3CL1 for recruiting macrophages to the neoplastic sites. Furthermore, multiple possible mechanisms may account for the anti-tumoral role of miR-125b, such as the following: (i) “miR-125b may enhance chromosomal stability by stimulating the expression of cohesin complex subunits (e.g., SMC2, SMC3, SMC4, STAG1, STAG2, SGO1, and PDS5B; referring to mRNA-Seq data)” (ii) “miR-125b may be transferred to tumor microenvironment via exosomes and inhibits TAM infiltration” (iii) “miR-125b may function through classical and/or nonclassical NF-kB signaling (e.g., RELB, NFKB2, and NFKBIA; referring to mRNA-Seq data)”.

Previous studies suggested that miR-125b plays a crucial role during the induced-differentiation of embryonic stem (ES) cells, without affecting their proliferation and self-renewal^48,49^. Consistent with these results, we found no effect of miR-125b on the proliferation and apoptosis of NCCIT human embryonic carcinoma cells (the malignant counterparts of ES cells) in vitro (Fig. 3). Whether miR-125b regulates other intrinsic properties of NCCIT tumor cells needs a further investigation.

It is unreliable to identify miRNA targets simply by prediction algorithms, like TargetScan^50^, and a combination approaches, such as transcriptome analysis, transcriptome-wide miRNA-mRNA interaction analysis, and cell-based screening systems, are suggested to be used^51–53^. In agree with this notion, we screen the differentially expressed genes among miR-125b antagomir-, control-, and miR-125b agomir-transfected tumor cells without bias; then, the gene lists are further filtered using several miRNA target prediction algorithms; and finally, CSF1 and CX3CL1 are confirmed to be direct targets of miR-125b by luciferase assays in this study.

A single miRNA may lack the power to drive significant biological effects; however, a combination of multiple miRNAs which target the core regulons is guaranteed^10,54^. In agree with this notion, we show that a set of miRNAs under miR-125b regulation are predicted to combinatorially target core regulons (e.g., CSF1, CX3CL1). However, the conclusion that miR-125b regulates CSF1 and CX3CL1 likely through a miRNA network of miRNAs was drawn by miRNA-seq and prediction algorithms; thus, further functional confirmation will be quite necessary. Future studies should highlight the miRNA-mRNA network in tumor progression, rather than exploring a single target gene per time. Similarly, the interference of single miRNA expression may have a limited inhibitory effect on tumors; thus, joint interference of multiple miRNAs with different or complementary mechanisms would be more effective for tumor treatment.

In conclusion, our study demonstrates that enforced miR-125b expression in NCCIT tumor cells can inhibit the production of tumor-derived chemokines (e.g., CSF1, CX3CL1), recruit less pro-tumorigenic macrophages to tumor sites, and ultimately suppress TGCT growth (Fig. 8). Our results provide a novel insight into the crosstalk between tumor cells and their microenvironment and raise the possibility of targeting miR-125b as miRNA therapeutics in TGCTs.

## Materials and Methods

### Reagents, cell culture, miRNAs

5-aza-2-deoxycytidine (5-aza), DPI, GSK126 and H_2_O_2_ were purchased from Sigma (St Louis, MO, USA). Human miR-125b antagomir, miR-125b agomir and control oligonucleotides were synthesized by GenePharma (Shanghai, China). The sequence of miR-125b antagomir, miR-125b agomir and control oligonucleotides was provided as follows: has-miR-125b antagomir: 5′-UCACAAGUUAGGGUCUCAGG GA-3′; has-miR-125b agomir: 5′-UCCCUGAGACCCUAACUUGUGA-3′ and 5′-ACAAGUUAGGGUCUCAGGGAUU-3′; antagomir NC: 5′-CAGUACUUUUGU GUAGUACAA-3′; and agomir NC: 5′-UUCUCCGAACGUGUCACGUTT-3′ and 5′-ACGUGACACGUUCGGAGAATT-3′. The human embryonic carcinoma cell line NCCIT and NTERA-2 cl.D1 [NT2/D1] were purchased from American Type Culture Collection (ATCC, VA, USA). NCCIT cells were cultured in RPMI 1640 (Invitrogen, Beijing, China) containing 10% fetal bovine serum (Invitrogen), 1% penicillin/stretomycin (Invitrogen) at 37 °C with 5% CO_2_. NCCIT cells were transfected with the miR-125b antagomir (50 nM) or miR-125b agomir (25 nM) or non-targeting control (NC) oligonucleotides (25 nM) using Entranster^TM^-R4000 transfection reagent (Engreen, Beijing, China). For drug treatment, cells were treated with 5-aza (5 µM, 72 h) or GSK126 (1000 nM, 72 h) or DPI (1.5 µM, 12 h) or H_2_O_2_ (25 µM, 4 h).

### Small interfering RNA (siRNA) transfection

For *CSF1* knockdown, NCCIT cells were transiently transfected with a pool of three siRNAs targeting human *CSF1* (814: 5′-GUCCGAACUUUCUAUGAGATT-3′ and 5′-UCUCAUAGAAAGUUCGGACTT-3′; 698: 5′-CCAUGCGCUUCAGAGAU AATT-3′ and 5′-UUAUCUCUGAAGCGCAUGGTT-3′; 569: 5′-GGCUGAUUGAC AGUCAGAUTT-3′ and 5′-AUCUGACUGUCAAUCAGCCTT-3′) or with nontargeting control siRNA (5′-UUCUCCGAACGUGUCACGUTT-3′ and 5′-ACGUGACACGUUCGGAGAATT-3′; all from GenePharma) using Entranster^TM^-R4000 (Engreen) at a final concentration of 0.1 μM. For *CX3CL1* knockdown, NCCIT cells were transiently transfected with a pool of three siRNAs (1086: 5′-CCUUAUCACUCCUGUCCCUTT-3′ and 5′-AGGGACAGGAGUGAUAAGGTT-3′; 819: 5′-GGAGAAUGCUCCGUCUGAA TT-3′ and 5′-UUCAGACGGAGCAUUCUCCTT-3′; 528: 5′-GACUCCUUCUUCCC AGGAATT-3′ and 5′-UUCCUGGGAAGAAGGAGUCTT-3′). Cells were harvested at 48 h after transfection.

### In vivo tumor xenograft models

All animal experiments were approved by the Institutional Animal Care and Use Committee (IACUC) of the Institute of Zoology, Chinese Academy of Sciences and in accordance with institutional and national guidelines. Model I: NCCIT cells were transfected with miR-125b antagomir or miR-125b agomir or control oligonucleotides and then implanted subcutaneously into the flanks of Balb/c nude mice (Charles River, Beijing, China). Model II: NCCIT xenografts of same size were chosen and then miR-125b antagomir (10 µg/xenograft) or miR-125b agomir (5 µg/xenograft) or control oligonucleotides (5 µg/xenograft) was injected into xenografts using Entranster^TM^ in vivo transfection reagent (Engreen) every week for two consecutive weeks.

### Clodronate treatment

Subcutaneous (s.c.) injection of miR-125b antagomir- or miR-125b agomir- or control oligonucleotide-transfected NCCIT cells was performed as described above. To induce host macrophage depletion, Balb/c nude mice were injected intraperitoneally (i.p.) with Clophosome^®^-clodronate liposome (anionic) or plain control liposome (anionic) (FormuMax Scientific, CA, USA) at a dose of 0.15 ml every six days after tumor inoculation for three weeks.

### Copy number analysis

Putative copy-number calls on 156 TGCT cases were collected from TCGA database and then were determined using GISTIC 2.0. Values: −2 = homozygous deletion; −1 = hemizygous deletion; 0 = neutral/no change; 1 = gain; 2 = high level amplification. cBio-Portal (http://cbioportal.org) was used to process copy number analysis as previously described^55^.

### Real-Time Quantitative RT-PCR

Total RNA was extracted from the harvested NCCIT cells or xenografts using miRcute miRNA Isolation Kit (Tiangen, Beijing, China). miRNAs from parafinized human tissue sections of seminoma and embryonic carcinoma (OriGene, Wuhan, China) was isolated miRNArep Pure FFPE Kit (Tiangen). Then, miRNAs were reversed transcribed with miRcute Plus miRNA First-Strand cDNA Synthesis Kit (Tiangen). Power SYBR Green PCR reactions were performed in triplicate for each sample using miRcute Plus miRNA qPCR Detection Kit (Tiangen). CT value of miR-125b was normalized to the value of U6 (Ambion, MS, USA). qRT-PCR analysis for *CSF1* and *CX3CL1* mRNA was performed as our previous description^56^.

### Histological staining

Xenograft tumor tissues were harvested and fixed in 4% paraformaldehyde (PFA) overnight. Five-micron paraffin-embedded sections were cut and stained with hematoxylin and eosin (Beyotime, Shanghai, China). Immunofluorescent staining was performed as previously described^57^. The rabbit monoclonal antibodies to Iba1 (ab178847) and CD163 (ab182422) were obtained from Abcam (Cambridge, MA, USA). Anti-rabbit fluorescent secondary antibody (Alexa Fluor488) was purchased from Cell Signaling Technology (Beverly, MA, USA). Nuclei were stained with DAPI (Beyotime).

### Cell proliferation assay

Cell proliferation assay was examined using the Cell Counting Kit-8 (CCK-8) Assay Kit (Beyotime). Cells were seeded into 96-well cell culture plates for each group, and assayed at 24 h, 48 h, and 72 h after transfection. CCK-8 was added into each well and the absorbance was read at 450 nm with a microplate spectrophotometer (Thermo Fisher Scientific, MS, USA).

### Apoptosis and cell cycle analysis

The amount of apoptosis was measured using the Annexin V-FITC/Propidium Iodide (PI) Apoptosis Detection Kit (Beyotime) and flow cytometry (BD FACSAria IIIu, CA, USA) according to the manufacturer’s instruction. The distribution of the G_0_/G_1_, S, and G_2_/M phases of the cell cycle was determined after cell fixation by flow cytometric analysis of PI-stained nuclei.

### EdU and TUNEL staining

EdU and TUNEL assays were performed using the BeyoClick^TM^ EdU Cell Proliferation Kit with Alexa Fluor 488 and Colorimetric TUNEL Apoptosis Assay Kit (all from Beyotime), respectively, in accordance with the manufacturer’s protocol. The number of EdU or TUNEL-positive cells was counted in six fields randomly, and the proliferation/apoptosis index for each field was calculated as the percent of positive cells relative to the total cells.

### RNA sequencing and miRNA sequencing

Total RNA (1 μg for each samples) was extracted from miR-125b antagomir-transfected, miR-125b agomir-transfected, and control oligonucleotide-transfected NCCIT cells using miRcute miRNA Isolation Kit (Tiangen, Beijing, China). Three duplicate samples for each treatment were included. Then, equal amounts of total RNA were analyzed using BGISEQ-500 platform and the sequencing procedure was performed by BGI (Shenzhen, China)^58^. The lists of differentially expressed genes or miRNAs (with an absolute value of fold change cut-off ≥ 1.3 or ≤ 0.75 and significance *p*-value < 0.05) were provided as Table S1 and Table S3, respectively. Enriched pathways were acquired by cluster analysis and listed in Table S2. For miRNA-mRNA network analysis, the target genes of differentially expressed miRNAs (from miRNA sequencing) were predicted using the TargetScan database, and then predicted target genes were further selected on the list of differentially expressed genes (from mRNA sequencing).

### Western blot

Western blot analysis was performed as described previously^59^. Briefly, proteins were electrophoresed in 10% SDS-PAGE gels and transferred to nitrocellulose membranes. The blots were blocked in 5% milk and incubated overnight at 4 °C with the primary antibodies, followed by incubation with anti-rabbit Dye 680CW or anti-mouse Dye 800CW (LI-COR, MO, USA) at 1/10,000 dilution for 1 h. The specific signals and the corresponding band intensities were evaluated using Odyssey Infrared Imaging system (Odyssey, Berlin, Germany). The protein level was normalized against GAPDH. The following antibodies were used in this study: rabbit anti-CSF1 (0.2 µg/ml; Abcam, ab9693), rabbit anti-CX3CL1 (1/5,000 dilution; Abcam, ab85034), and mouse anti-GAPDH (1/10,000 dilution; Bioworld, MB001).

### Luciferase assays

The wild-type and mutant 3’ UTR of human *CSF1* (~800 bp) and *CX3CL1* (~1700 bp) were individually inserted into the pMirTarget (OriGene, CA, USA) using the EcoR1 and Xba1 sites. NCCIT cells were maintained at approximately 50% confluence and co-transfected with wild-type or mutant 3’ UTR luciferase plasmid (0.2 μg) and miR-125b mimic or mimic negative control (50 nM; Qiagen, Berlin, Germany) using Lipofectamine 2000 (Invitrogen, CA, USA). After 48 h of incubation, luciferase activity was assayed using Steady-Glo Luciferase Assay System (Promega, WI, USA). Renilla luciferase activity was co-transfected and served as a control for transfection efficiency.

### Enzyme-linked immunosorbent assay (ELISA)

Human CSF1 and CX3CL1 concentrations from cell lysates and cell culture supernatant were measured using CSF1 ELISA Kit (Abnova, CA, USA, KA0384) and CX3CL1 ELISA Kit (Abnova, KA0545), respectively, following the manufacturer’s instructions. Concentrations (pg/ml) were determined by absorbance measurements at 450 nm against a standard curve in a competitive assay using an ELISA reader (DeTie, Nanjing, China, HBS-1096A).

### Statistics

Data were compared for statistical significance using GraphPad Prism version 5.01 (Graph Pad Software Inc., CA, USA). Student’s *t* tests (for two groups) and one-way ANOVA (for three or more groups) were used for the analyses. The data were presented as the mean ± SEM of at least three independent experiments, and differences were considered statistically significant at *p < 0.05, **p < 0.01, and ***p < 0.0001.

## Electronic supplementary material

Figure S1, Figure S2, Figure S3, Table S1

Table S2

Table S3

supplementary figure legends

Fig.S1

Fig.S2

Fig.S3
