# Supplementary material for: A miR-125b/CSF1-CX3CL1/tumor-associated macrophage recruitment axis controls testicular germ cell tumor growth
Source: Cell Death Dis. 2018 Sep 20;9(10):962. doi: 10.1038/s41419-018-1021-z (PMC6148032; doi:10.1038/s41419-018-1021-z)
Supplement: Supplementary file 1 — Figure S1, Figure S2, Figure S3, Table S1 [file 41419_2018_1021_MOESM1_ESM.docx]

| **Table S1. Differentially expressed genes among miR-125b agomir (ago)-, miR-125b antagomir (ant)-, and negative control (NC)-transfected NCCIT tumor cells by RNA sequencing.** | | | | | | | | | | |
| --- | --- | --- | --- | --- | --- | --- | --- | --- | --- | --- |
|  | Symbol | ago-1_FPKM | NC-1_FPKM | ant-1_FPKM | ago-2_FPKM | NC-2_FPKM | ant-2_FPKM | ago-3_FPKM | NC-3_FPKM | ant-3_FPKM |
| **Cluster 1** | IRF1 | 16.99 | 21.34 | 33.4 | 13.58 | 19.31 | 54.4 | 17.26 | 40.79 | 55.36 |
|  | CX3CL1 | 0.42 | 0.96 | 1.16 | 0.57 | 0.72 | 2.97 | 0.83 | 2.1 | 2.66 |
|  | CD83 | 1.44 | 2.18 | 2.22 | 1.8 | 2.33 | 7.36 | 1.85 | 3.96 | 4.73 |
|  | CSF1 | 0.6 | 0.82 | 1.51 | 0.35 | 0.43 | 1.71 | 0.38 | 1.06 | 1.24 |
|  | BCL3 | 8.3 | 9.13 | 11.37 | 6.7 | 8.69 | 29.08 | 7.28 | 17.12 | 20.25 |
|  | SERPINA3 | 2.34 | 2.52 | 2.83 | 1.27 | 1.44 | 4.07 | 1.38 | 2.88 | 3.74 |
|  | JUNB | 43.21 | 54.64 | 65.23 | 36.97 | 46.89 | 144.87 | 53.32 | 89.47 | 105.57 |
| **Cluster 2** | PHF3 | 2.37 | 1.71 | 0.62 | 2.46 | 1.61 | 0.89 | 1.76 | 1.52 | 0.68 |
|  | NDC80 | 4.95 | 3.37 | 1.98 | 5.71 | 4.07 | 2.43 | 4.88 | 3.69 | 2.42 |
|  | NAA15 | 4.02 | 2.45 | 1.19 | 5.14 | 3.32 | 2.87 | 3.75 | 3.22 | 2.22 |
|  | APC | 1.06 | 0.63 | 0.26 | 1.08 | 0.52 | 0.39 | 0.62 | 0.53 | 0.39 |
|  | NPAT | 1.45 | 0.78 | 0.35 | 1.44 | 1.04 | 0.67 | 1.16 | 0.98 | 0.52 |
|  | PLS3 | 7.4 | 5.03 | 2.2 | 9.25 | 5.62 | 4.43 | 6.86 | 5.24 | 3.74 |
|  | ZFYVE16 | 0.67 | 0.47 | 0.14 | 0.8 | 0.42 | 0.38 | 0.58 | 0.51 | 0.25 |
|  | AHCTF1 | 2.16 | 1.4 | 0.63 | 2.06 | 1.19 | 1.03 | 1.56 | 1.31 | 0.88 |
|  | U2AF1L5 | 19.51 | 18.99 | 30.48 | 8.35 | 22.67 | 24.9 | 8.28 | 19.34 | 37.96 |
|  | ARHGAP18 | 1.16 | 0.66 | 0.24 | 1.05 | 0.58 | 0.51 | 0.75 | 0.7 | 0.47 |
|  | AHI1 | 0.76 | 0.37 | 0.18 | 0.59 | 0.43 | 0.26 | 0.37 | 0.29 | 0.18 |
|  | KIF14 | 0.97 | 0.52 | 0.26 | 0.97 | 0.64 | 0.37 | 0.65 | 0.5 | 0.36 |
|  | PIK3CA | 1.21 | 0.77 | 0.23 | 1.2 | 0.77 | 0.63 | 0.86 | 0.79 | 0.42 |
|  | PLK4 | 1.78 | 1.17 | 0.35 | 2.12 | 1.35 | 1.17 | 1.73 | 1.24 | 0.89 |
|  | PDS5B | 2.47 | 1.5 | 0.88 | 3.13 | 2 | 1.4 | 2.24 | 1.78 | 1.11 |
|  | RELB | 10.34 | 12.18 | 15.6 | 11.86 | 11.74 | 40.75 | 11.42 | 29.4 | 34.22 |
|  | ZFP37 | 0.23 | 0.17 | 0.02 | 0.33 | 0.14 | 0.1 | 0.19 | 0.09 | 0.1 |
|  | TTC3 | 9.03 | 7.08 | 2.49 | 10.78 | 7.14 | 3.75 | 7.53 | 5.64 | 3.27 |
|  | NFKB2 | 22.18 | 21.87 | 23.87 | 18.63 | 18.63 | 50.36 | 17.99 | 40.07 | 33.65 |
|  | VPS50 | 1.75 | 1.19 | 0.64 | 1.5 | 0.86 | 0.31 | 1.61 | 1.44 | 0.98 |
|  | ZNF638 | 2.96 | 2.04 | 0.69 | 3.01 | 2.2 | 1.18 | 2.12 | 1.53 | 0.85 |
|  | CFAP44 | 0.1 | 0.08 | 0.04 | 0.19 | 0.07 | 0.02 | 0.18 | 0.08 | 0.03 |
|  | ZNF816 | 1.32 | 0.6 | 0.22 | 0.91 | 0.62 | 0.47 | 0.84 | 0.68 | 0.43 |
|  | FER | 0.97 | 0.75 | 0.25 | 1.69 | 1.23 | 0.81 | 1.21 | 0.71 | 0.37 |
|  | PHIP | 1.04 | 0.69 | 0.25 | 1.35 | 0.93 | 0.46 | 0.88 | 0.62 | 0.32 |
|  | RAD51AP1 | 4.95 | 3.66 | 1.9 | 5.64 | 3.82 | 3.2 | 4.73 | 3.57 | 2.51 |
|  | NOL8 | 3.6 | 2.46 | 0.93 | 4.01 | 2.91 | 1.31 | 2.53 | 1.83 | 1.23 |
|  | KIF15 | 1.68 | 1.08 | 0.54 | 1.76 | 1.11 | 0.65 | 1.27 | 0.96 | 0.6 |
|  | STAG2 | 6.1 | 4.03 | 1.21 | 7.79 | 4.29 | 3.26 | 5.24 | 5 | 2.83 |
|  | WDHD1 | 1.15 | 0.92 | 0.32 | 1.26 | 0.72 | 0.56 | 1.03 | 0.68 | 0.54 |
|  | TOPORS | 1.32 | 1.17 | 0.54 | 2.05 | 1.19 | 0.98 | 1.38 | 0.97 | 0.75 |
|  | SMC2 | 2.35 | 1.74 | 0.7 | 3.29 | 2.02 | 1.01 | 2.37 | 1.89 | 0.82 |
|  | ANLN | 11.93 | 8.13 | 3.91 | 16.37 | 9.22 | 8.09 | 12.96 | 10.13 | 7.54 |
|  | CRYBG3 | 0.4 | 0.21 | 0.08 | 0.24 | 0.21 | 0.15 | 0.23 | 0.17 | 0.11 |
|  | CENPU | 3.5 | 1.79 | 1.21 | 4.02 | 2.87 | 1.68 | 2.49 | 2.25 | 1.08 |
|  | ZC3H12A | 7.41 | 5.68 | 6.01 | 4.91 | 4.42 | 34.54 | 5.64 | 19.16 | 15.71 |
|  | ZGRF1 | 0.28 | 0.19 | 0.09 | 0.26 | 0.18 | 0.1 | 0.28 | 0.15 | 0.09 |
|  | ERGIC2 | 4.29 | 3.19 | 1.29 | 6.59 | 3.87 | 2.12 | 4.91 | 3.14 | 1.93 |
|  | ZNF225 | 0.4 | 0.25 | 0.1 | 0.46 | 0.23 | 0.17 | 0.38 | 0.29 | 0.21 |
|  | STXBP3 | 2.85 | 1.81 | 0.76 | 3.1 | 1.67 | 1.53 | 2.21 | 1.9 | 1.29 |
|  | HPGD | 6.72 | 4.08 | 2.34 | 7.51 | 5.4 | 3.87 | 4.96 | 4.27 | 3.27 |
|  | ZNF268 | 0.58 | 0.42 | 0.14 | 0.97 | 0.47 | 0.33 | 0.56 | 0.45 | 0.29 |
|  | MRPL1 | 3.6 | 3.18 | 1.45 | 5.15 | 3.51 | 2.08 | 4.11 | 2.45 | 1.69 |
|  | SREK1IP1 | 0.96 | 0.56 | 0.37 | 1.24 | 1 | 0.56 | 1.01 | 0.65 | 0.5 |
|  | SASS6 | 0.71 | 0.41 | 0.23 | 0.82 | 0.41 | 0.36 | 0.5 | 0.43 | 0.24 |
|  | LTA | 1.12 | 1.74 | 2.58 | 1.09 | 1.85 | 2.21 | 1.32 | 1.93 | 2.46 |
|  | USP15 | 1.09 | 0.69 | 0.23 | 1.05 | 0.49 | 0.38 | 0.62 | 0.58 | 0.46 |
|  | RECQL | 4.04 | 2.73 | 1.09 | 4.36 | 3.01 | 2.06 | 3.92 | 2.62 | 1.69 |
|  | WDR75 | d | 6.32 | 2.67 | 10.2 | 6.39 | 6.31 | 7.94 | 5.74 | 4.24 |
|  | TMTC3 | 0.44 | 0.25 | 0.05 | 0.6 | 0.29 | 0.24 | 0.51 | 0.42 | 0.24 |
|  | SLK | 2.59 | 1.44 | 0.67 | 2.77 | 1.61 | 1.26 | 1.83 | 1.66 | 0.94 |
|  | RIF1 | 1.74 | 1.01 | 0.39 | 1.5 | 0.85 | 0.68 | 1.33 | 1.24 | 0.64 |
|  | DBF4 | 7.18 | 5.58 | 2.81 | 11.41 | 7.23 | 5.77 | 8.91 | 6.22 | 4.86 |
|  | TOP2B | 5.47 | 3.74 | 1.37 | 7.91 | 5.05 | 3.37 | 5.31 | 4.41 | 2.78 |
|  | NEMF | 1.29 | 0.94 | 0.4 | 2.08 | 1.46 | 0.55 | 1.32 | 0.85 | 0.41 |
|  | DOCK11 | 2.23 | 1.44 | 0.56 | 2.47 | 1.49 | 0.72 | 1.39 | 1.29 | 0.69 |
|  | SPDL1 | 2.51 | 1.76 | 0.77 | 3.47 | 2.7 | 1.49 | 2.48 | 1.37 | 1.13 |
|  | BLM | 2.11 | 1.56 | 0.77 | 2.93 | 1.99 | 1.45 | 2.06 | 1.36 | 1.16 |
|  | RLF | 1.04 | 0.52 | 0.27 | 1.13 | 0.68 | 0.52 | 0.85 | 0.69 | 0.35 |
|  | ZWILCH | 2.73 | 1.92 | 0.98 | 4.11 | 2.61 | 2.08 | 3.4 | 2.61 | 1.28 |
|  | MIS18BP1 | 1.26 | 0.88 | 0.29 | 1.41 | 1.01 | 0.56 | 1.07 | 0.6 | 0.33 |
|  | MTUS1 | 2.26 | 1.49 | 1.02 | 2.1 | 1.22 | 0.81 | 1.64 | 1.43 | 0.97 |
|  | TOP2A | 12.49 | 8.59 | 3.65 | 18.39 | 11.96 | 4.63 | 12.48 | 8.17 | 4.4 |
|  | ZNF248 | 1.54 | 1.37 | 0.99 | 1.66 | 1.3 | 1.22 | 1.36 | 1.2 | 1.14 |
|  | SMC3 | 3.97 | 2.53 | 0.9 | 4.54 | 3.28 | 1.52 | 3.18 | 2.3 | 1.38 |
|  | SGO2 | 1.27 | 0.71 | 0.35 | 1.46 | 0.97 | 0.49 | 1.08 | 0.67 | 0.54 |
|  | USPL1 | 2.35 | 1.54 | 0.71 | 2.34 | 1.77 | 1.29 | 2.24 | 1.75 | 1.02 |
|  | TTK | 4.36 | 3.13 | 0.94 | 5.25 | 3.47 | 1.89 | 3.92 | 1.89 | 1.43 |
|  | SUCO | 2.05 | 1.04 | 0.45 | 1.66 | 1.17 | 1.13 | 1.49 | 1.17 | 0.97 |
|  | CCDC82 | 2.65 | 1.61 | 0.83 | 2.81 | 1.6 | 1.16 | 2.37 | 2.13 | 0.85 |
|  | HSP90AA1 | 112.61 | 98.47 | 36.59 | 176.99 | 125.8 | 45.84 | 124.81 | 68.68 | 40.55 |
|  | KNTC1 | 2.31 | 1.58 | 0.56 | 2.54 | 1.8 | 1.22 | 2.12 | 1.39 | 1.02 |
|  | NOC3L | 2.38 | 1.41 | 0.62 | 2.09 | 1.16 | 1.14 | 1.95 | 1.71 | 1.15 |
|  | HAT1 | 14.17 | 10.04 | 4.89 | 17.74 | 12.61 | 7.67 | 14.2 | 9.26 | 6.37 |
|  | RBM41 | 1.75 | 0.8 | 0.38 | 1.28 | 0.69 | 0.5 | 0.83 | 0.54 | 0.38 |
|  | ORC3 | 5.42 | 3.69 | 1.84 | 6.22 | 4.5 | 3.14 | 4.9 | 3.25 | 2.37 |
|  | ROCK2 | 6.93 | 4.22 | 1.72 | 6.06 | 4.2 | 2.68 | 4.56 | 4.24 | 2.3 |
|  | ZNF117 | 0.16 | 0.13 | 0.09 | 0.29 | 0.2 | 0.05 | 0.24 | 0.11 | 0.07 |
|  | CCDC88A | 0.97 | 0.62 | 0.28 | 1.26 | 0.63 | 0.42 | 0.69 | 0.67 | 0.31 |
|  | C12orf4 | 2.06 | 1.15 | 0.43 | 2.8 | 1.67 | 1.33 | 1.74 | 1.56 | 0.76 |
|  | SMC4 | 12.21 | 9.51 | 4.01 | 15.9 | 10.72 | 5.08 | 11.06 | 7.17 | 4.03 |
|  | ZNF189 | 2.21 | 1.57 | 0.72 | 2.18 | 1.44 | 1.24 | 1.69 | 1.37 | 1.05 |
|  | RPAP3 | 4.12 | 2.7 | 1.05 | 4.45 | 3.06 | 1.88 | 3.13 | 2.64 | 1.86 |
|  | HMMR | 4.14 | 2.63 | 1.23 | 5.46 | 3.05 | 1.73 | 3.7 | 2.71 | 1.39 |
|  | GCC2 | 1.28 | 0.9 | 0.37 | 1.68 | 0.83 | 0.55 | 1.07 | 0.88 | 0.54 |
|  | ARAP2 | 0.55 | 0.27 | 0.1 | 0.5 | 0.21 | 0.25 | 0.35 | 0.37 | 0.19 |
|  | STAG1 | 4.1 | 2.71 | 1.05 | 4.8 | 3.09 | 2.15 | 3.78 | 3.39 | 2.39 |
|  | TOPBP1 | 7.47 | 4.99 | 2 | 7.94 | 5.72 | 3.41 | 6.66 | 4.5 | 3.17 |
|  | COPS2 | 3.4 | 2.22 | 1.15 | 5.08 | 3.19 | 2.05 | 3.87 | 2.96 | 1.78 |
|  | CENPC | 0.59 | 0.38 | 0.18 | 0.78 | 0.39 | 0.27 | 0.68 | 0.41 | 0.27 |
|  | TMF1 | 1.67 | 0.87 | 0.31 | 1.47 | 0.99 | 0.76 | 1.29 | 0.99 | 0.48 |
|  | RB1CC1 | 1.95 | 1.16 | 0.63 | 2.1 | 1.23 | 0.81 | 1.71 | 1.36 | 0.64 |
|  | ASPM | 1.2 | 1.1 | 0.33 | 1.69 | 0.95 | 0.69 | 1.07 | 0.73 | 0.53 |
|  | OXR1 | 17.83 | 10.18 | 5.17 | 16.45 | 9.42 | 8.2 | 12.78 | 11.66 | 8.1 |
|  | EMC2 | 1.09 | 0.83 | 0.61 | 1.5 | 0.99 | 0.6 | 1.35 | 0.96 | 0.6 |
|  | KRTAP2-1 | 0.74 | 1.17 | 1.49 | 0.77 | 1.45 | 3.77 | 1.07 | 3.04 | 4.04 |
|  | PNPLA8 | 1.25 | 0.58 | 0.38 | 1.41 | 0.81 | 0.7 | 1.03 | 0.82 | 0.49 |
|  | BORA | 2.99 | 2.22 | 1.02 | 4.17 | 2.78 | 2.02 | 2.93 | 1.99 | 1.49 |
|  | KIF11 | 3.46 | 2.72 | 1.25 | 5.81 | 4.03 | 2.35 | 4.56 | 2.86 | 2.42 |
|  | IFT80 | 1.98 | 1.23 | 0.38 | 1.57 | 0.83 | 0.54 | 1.62 | 1.14 | 0.58 |
|  | CCDC150 | 0.28 | 0.12 | 0.08 | 0.41 | 0.14 | 0.03 | 0.23 | 0.12 | 0.09 |
|  | KIAA1524 | 1.65 | 0.81 | 0.49 | 1.64 | 1.08 | 0.71 | 1.2 | 1 | 0.63 |
|  | RGPD5 | 2.61 | 1.87 | 0.63 | 3.05 | 1.95 | 1.26 | 2.15 | 1.64 | 1.24 |
|  | NEURL3 | 0.65 | 0.65 | 0.88 | 0.54 | 0.74 | 2.17 | 0.53 | 1.18 | 2.08 |
|  | ATAD2 | 6 | 3.64 | 1.32 | 5.76 | 3.68 | 3.15 | 4.53 | 4.14 | 2.7 |
|  | AGTPBP1 | 3.46 | 2.44 | 0.93 | 3.29 | 2.05 | 1.71 | 2.62 | 2.3 | 1.69 |
|  | CCDC66 | 1.15 | 0.8 | 0.41 | 0.92 | 0.59 | 0.4 | 0.87 | 0.67 | 0.41 |
|  | NFKBIA | 11.31 | 11.1 | 12.63 | 10.85 | 9.98 | 80.38 | 9.47 | 43.99 | 37.48 |
|  | C11orf96 | 4.1 | 6.54 | 8.48 | 4.87 | 6.06 | 13.6 | 6.61 | 11.92 | 15.73 |
|  | RAET1L | 2.34 | 2.12 | 1.64 | 1.06 | 1 | 5.73 | 0.74 | 4.96 | 4.46 |
|  | ZCCHC6 | 2.21 | 1.42 | 0.61 | 1.74 | 1.08 | 0.8 | 1.64 | 1.34 | 0.79 |
|  | PLCB4 | 0.96 | 0.66 | 0.2 | 0.9 | 0.4 | 0.31 | 0.7 | 0.6 | 0.36 |
|  | STAT5A | 2.7 | 3.33 | 5.17 | 2.63 | 3.65 | 8.54 | 2.55 | 5.5 | 7.79 |
|  | CENPE | 0.34 | 0.26 | 0.07 | 0.51 | 0.25 | 0.1 | 0.3 | 0.23 | 0.1 |
|  | ETAA1 | 0.96 | 0.77 | 0.37 | 1.34 | 0.75 | 0.47 | 0.94 | 0.57 | 0.36 |
|  | AIMP1 | 4.94 | 3.5 | 2.26 | 5.79 | 4.22 | 2.82 | 4.57 | 2.9 | 2.52 |
|  | USP16 | 3.2 | 2.41 | 0.91 | 5.52 | 2.98 | 1.4 | 3.17 | 2.84 | 1.61 |
|  | ZNF235 | 0.38 | 0.37 | 0.13 | 0.66 | 0.29 | 0.25 | 0.59 | 0.24 | 0.13 |
