# Supplementary material for: A miR-125b/CSF1-CX3CL1/tumor-associated macrophage recruitment axis controls testicular germ cell tumor growth
Source: Cell Death Dis. 2018 Sep 20;9(10):962. doi: 10.1038/s41419-018-1021-z (PMC6148032; doi:10.1038/s41419-018-1021-z)
Supplement: Supplementary file 2 — Table S2 [file 41419_2018_1021_MOESM2_ESM.docx]

| **Table S2. Cluster analysis revealed the top enriched pathways under the regulation of miR-125b.** | | | |
| --- | --- | --- | --- |
| **Enriched pathways** | | **Differentially expressed genes** | **# of genes** |
| **Immune response** | Macrophage recruitment pathway | *CX3CL1*, *CD83*, *CSF1*, *LTA*, *AIMP1* | 5 |
|  | NF-kB signaling | *RELB*, *NFKB2*, *NFKBIA* | 3 |
|  | Others | *IRF1*,  *BCL3*, *SERPINA3*, *JunB*, *ZC3H12A*, *ZNF268*, *HMMR*, *RAET1L* | 8 |
| **Cell cycle** | Centromere protein | *CENPU*, *CENPC*, *CENPE* | 3 |
|  | Others | *NDC80*, *PLK4*, *DBF4*, *SPDL1*, *ZWILCH*, *MIS18BP1*, *TTK*, *KNTC1*, *ORC3*, *RB1CC1*, *ASPM*, *BORA*, *KIF11* | 13 |
| **Transcription & Modification** |  | *NAA15*, *NPAT*, *ZFP37*, *ZNF638*, *RAD51AP1*, *RECQL*, *RIF1*, *TOP2B*, *BLM*, *TOP2A*, *HAT1*, *RPAP3*, *TOPBP1*, *COPS2*, *ZCCHC6* | 15 |
| **Signal transduction** |  | *APC*, *ZFYVE16*, *PIK3CA*, *FER*, *PHIP*, *HPGD*, *SLK*, *MTUS1*, *HSP90AA*, *ROCK2*, *OXR1*, *PLCB4*, *STAT5A* | 13 |
| **Cytoskeleton** |  | *PLS3*, *ARHGAP18*, *KIF14*, *KIF15*, *ANLN*, *ARAP2* | 6 |
| **Ubiquitination** |  | *TTC3*, *TOPORS*, *USP15*, *USPL1*, *TMF1*, *NEURL3*, *USP16* | 7 |
| **Cohesin in Cancer** |  | *SMC2*, *SMC3*, *SMC3*, *STAG1*, *STAG2*, *SGO2*, *PDS5B* | 7 |
