# Supplementary material for: A miR-125b/CSF1-CX3CL1/tumor-associated macrophage recruitment axis controls testicular germ cell tumor growth
Source: Cell Death Dis. 2018 Sep 20;9(10):962. doi: 10.1038/s41419-018-1021-z (PMC6148032; doi:10.1038/s41419-018-1021-z)
Supplement: Supplementary file 3 — Table S3 [file 41419_2018_1021_MOESM3_ESM.docx]

**Table S3. Differentially expressed miRNAs among miR-125b agomir (ago)-, miR-125b antagomir (ant)-, and negative control (NC)-transfected NCCIT tumor cells by miRNA sequencing**

|  | | | | | | | | | | |  |  |  |
| --- | --- | --- | --- | --- | --- | --- | --- | --- | --- | --- | --- | --- | --- |
|  | Symbol | Read count (NC) | Read count (ago) | Expression (NC) | Expression (ago) | **Fold Change(ago/NC)** | P value | Read count (NC) | Read count (ant) | Expression (NC) | Expression (ant) | **Fold Change(ant/NC)** | P value |
|  | novel_mir6 | 74 | 437 | 0.85 | 5.57 | **6.92** | 6.44E-07 | 74 | 53 | 0.85 | 0.60 | **0.72** | 9.12E-07 |
| **Cluster 1** | miR-378e | 2565 | 12180 | 29.54 | 158.16 | **5.57** | 1.02E-11 | 2565 | 942 | 29.54 | 10.69 | **0.37** | 1.18E-13 |
|  | miR-409 | 4 | 18 | 0.04 | 0.22 | **5.28** | 3.06E-05 | 4 | 1 | 0.04 | 0.01 | **0.25** | 2.63E-07 |
|  | miR-4792 | 16 | 66 | 0.18 | 0.83 | **4.84** | 5.50E-07 | 16 | 12 | 0.18 | 0.14 | **0.75** | 3.12E-08 |
|  | novel_mir117 | 11 | 36 | 0.13 | 0.42 | **3.84** | 1.13E-07 | 11 | 2 | 0.13 | 0.01 | **0.05** | 6.39E-07 |
|  | miR-6875 | 2 | 5 | 0.02 | 0.06 | **2.93** | 9.91E-05 | 2 | 1 | 0.02 | 0.01 | **0.50** | 8.59E-04 |
|  | miR-4664 | 326 | 712 | 3.68 | 8.74 | **2.56** | 1.08E-09 | 326 | 237 | 3.68 | 2.69 | **0.73** | 3.18E-09 |
|  | novel_mir344 | 43 | 88 | 0.50 | 1.08 | **2.40** | 6.39E-07 | 43 | 31 | 0.50 | 0.35 | **0.73** | 3.61E-07 |
|  | novel_mir400 | 22 | 45 | 0.25 | 0.53 | **2.40** | 3.61E-07 | 22 | 15 | 0.25 | 0.17 | **0.69** | 2.05E-07 |
|  | miR-4780 | 3 | 6 | 0.04 | 0.07 | **2.34** | 6.78E-04 | 3 | 1 | 0.04 | 0.01 | **0.34** | 5.38E-06 |
|  | miR-7704 | 762 | 1368 | 8.67 | 17.11 | **2.11** | 1.79E-09 | 762 | 563 | 8.67 | 6.39 | **0.74** | 1.17E-10 |
|  | novel_mir87 | 12 | 21 | 0.13 | 0.24 | **2.05** | 2.19E-06 | 12 | 1 | 0.13 | 0.01 | **0.04** | 2.47E-05 |
|  | miR-6755 | 6 | 10 | 0.07 | 0.12 | **1.95** | 2.35E-06 | 6 | 3 | 0.07 | 0.03 | **0.50** | 3.54E-05 |
|  | miR-3679 | 6 | 10 | 0.07 | 0.12 | **1.95** | 4.42E-06 | 6 | 3 | 0.07 | 0.03 | **0.50** | 6.18E-05 |
|  | miR-6772 | 3 | 5 | 0.03 | 0.06 | **1.95** | 1.58E-05 | 3 | 1 | 0.03 | 0.01 | **0.34** | 1.08E-04 |
|  | miR-4306 | 3 | 5 | 0.03 | 0.07 | **1.95** | 1.25E-05 | 3 | 2 | 0.03 | 0.02 | **0.67** | 5.98E-05 |
|  | miR-4753 | 3 | 5 | 0.03 | 0.06 | **1.95** | 3.01E-05 | 3 | 2 | 0.03 | 0.02 | **0.67** | 1.06E-04 |
|  | miR-211 | 14 | 21 | 0.16 | 0.25 | **1.76** | 5.78E-07 | 14 | 6 | 0.16 | 0.07 | **0.43** | 1.06E-06 |
|  | miR-765 | 6 | 9 | 0.07 | 0.11 | **1.76** | 1.12E-04 | 6 | 4 | 0.07 | 0.05 | **0.67** | 1.90E-06 |
|  | miR-6797 | 2 | 3 | 0.02 | 0.04 | **1.76** | 3.29E-05 | 2 | 1 | 0.02 | 0.01 | **0.50** | 6.13E-05 |
|  | miR-506 | 2 | 3 | 0.02 | 0.04 | **1.76** | 2.17E-04 | 2 | 1 | 0.02 | 0.01 | **0.50** | 1.11E-05 |
|  | miR-4505 | 2 | 3 | 0.02 | 0.04 | **1.76** | 6.20E-05 | 2 | 1 | 0.02 | 0.01 | **0.50** | 2.01E-05 |
|  | miR-378d | 127605 | 177672 | 1422.04 | 2025.90 | **1.63** | 5.28E-12 | 127605 | 173 | 1422.04 | 1.96 | **0.01** | 3.67E-11 |
|  | novel_mir331 | 46 | 62 | 0.52 | 0.74 | **1.58** | 1.44E-08 | 46 | 35 | 0.52 | 0.40 | **0.77** | 8.40E-07 |
|  | miR-6742 | 3 | 4 | 0.03 | 0.05 | **1.56** | 6.44E-05 | 3 | 1 | 0.03 | 0.01 | **0.34** | 3.67E-05 |
|  | miR-6819 | 20 | 26 | 0.23 | 0.32 | **1.52** | 5.93E-08 | 20 | 15 | 0.23 | 0.17 | **0.75** | 6.72E-07 |
|  | novel_mir349 | 10 | 13 | 0.12 | 0.16 | **1.52** | 1.26E-05 | 10 | 2 | 0.12 | 0.01 | **0.05** | 7.79E-05 |
|  | miR-4797 | 7 | 9 | 0.08 | 0.11 | **1.51** | 1.27E-04 | 7 | 3 | 0.08 | 0.03 | **0.43** | 2.96E-05 |
|  | miR-365a | 64 | 81 | 0.74 | 0.96 | **1.48** | 2.25E-08 | 64 | 41 | 0.74 | 0.47 | **0.64** | 2.14E-06 |
|  | miR-371b | 1573 | 1936 | 18.46 | 24.27 | **1.44** | 4.54E-11 | 1573 | 703 | 18.46 | 7.96 | **0.45** | 1.96E-09 |
|  | novel_mir80 | 36 | 44 | 0.41 | 0.54 | **1.43** | 4.85E-07 | 36 | 16 | 0.41 | 0.18 | **0.45** | 1.13E-07 |
|  | novel_mir54 | 88 | 106 | 1.02 | 1.24 | **1.41** | 6.99E-07 | 88 | 30 | 1.02 | 0.34 | **0.34** | 4.12E-08 |
|  | novel_mir177 | 10 | 12 | 0.12 | 0.16 | **1.41** | 5.16E-05 | 10 | 1 | 0.12 | 0.01 | **0.05** | 5.97E-05 |
|  | miR-4762 | 5 | 6 | 0.06 | 0.07 | **1.41** | 2.86E-09 | 5 | 2 | 0.06 | 0.02 | **0.40** | 6.81E-05 |
|  | miR-4799 | 15 | 17 | 0.17 | 0.21 | **1.33** | 6.06E-08 | 15 | 11 | 0.17 | 0.12 | **0.74** | 1.47E-09 |
|  | miR-345 | 8 | 9 | 0.09 | 0.11 | **1.32** | 6.36E-05 | 8 | 3 | 0.09 | 0.03 | **0.38** | 2.34E-04 |
| **Cluster 2** | miR-516b | 6 | 3 | 0.07 | 0.04 | **0.59** | 1.29E-06 | 6 | 17 | 0.07 | 0.19 | **2.85** | 9.79E-04 |
|  | miR-4765 | 4 | 2 | 0.05 | 0.02 | **0.59** | 2.94E-05 | 4 | 8 | 0.05 | 0.09 | **2.01** | 3.00E-04 |
|  | miR-214 | 2 | 1 | 0.02 | 0.01 | **0.59** | 5.08E-05 | 2 | 12 | 0.02 | 0.14 | **6.03** | 4.53E-05 |
|  | miR-518e | 2 | 1 | 0.02 | 0.01 | **0.59** | 9.30E-04 | 2 | 6 | 0.02 | 0.07 | **3.02** | 4.54E-04 |
|  | miR-6716 | 2 | 1 | 0.02 | 0.01 | **0.59** | 1.05E-07 | 2 | 5 | 0.02 | 0.06 | **2.51** | 3.59E-05 |
|  | miR-4701 | 2 | 1 | 0.02 | 0.01 | **0.59** | 4.12E-05 | 2 | 5 | 0.02 | 0.06 | **2.51** | 9.62E-04 |
|  | miR-6845 | 2 | 1 | 0.02 | 0.01 | **0.59** | 1.67E-05 | 2 | 7 | 0.02 | 0.08 | **3.52** | 2.23E-04 |
|  | miR-4784 | 7 | 3 | 0.08 | 0.03 | **0.50** | 4.76E-04 | 7 | 14 | 0.08 | 0.16 | **2.01** | 1.41E-05 |
|  | miR-6767 | 7 | 3 | 0.08 | 0.03 | **0.50** | 4.12E-06 | 7 | 14 | 0.08 | 0.16 | **2.01** | 1.75E-05 |
|  | miR-6763 | 5 | 2 | 0.06 | 0.03 | **0.47** | 1.67E-06 | 5 | 17 | 0.06 | 0.19 | **3.42** | 4.24E-04 |
|  | miR-3136 | 5 | 2 | 0.06 | 0.02 | **0.47** | 1.26E-07 | 5 | 10 | 0.06 | 0.12 | **2.01** | 7.60E-04 |
|  | miR-4750 | 3 | 1 | 0.04 | 0.01 | **0.39** | 3.94E-06 | 3 | 7 | 0.04 | 0.08 | **2.35** | 7.52E-04 |
|  | miR-4742 | 3 | 1 | 0.04 | 0.01 | **0.39** | 8.75E-05 | 3 | 8 | 0.04 | 0.09 | **2.68** | 1.39E-06 |
|  | miR-6800 | 3 | 1 | 0.04 | 0.01 | **0.39** | 2.93E-05 | 3 | 7 | 0.04 | 0.08 | **2.35** | 3.85E-04 |
|  | miR-3687 | 95 | 28 | 1.08 | 0.33 | **0.35** | 3.95E-10 | 95 | 204 | 1.08 | 2.32 | **2.16** | 1.01E-09 |
|  | miR-6757 | 11 | 3 | 0.13 | 0.03 | **0.32** | 2.63E-06 | 11 | 22 | 0.13 | 0.25 | **2.01** | 2.37E-06 |
|  | miR-6848 | 4 | 1 | 0.05 | 0.01 | **0.29** | 4.11E-05 | 4 | 9 | 0.05 | 0.10 | **2.26** | 2.53E-05 |
|  | miR-3935 | 4 | 1 | 0.05 | 0.01 | **0.29** | 3.22E-05 | 4 | 9 | 0.05 | 0.10 | **2.26** | 1.07E-04 |
|  | novel_mir370 | 41 | 10 | 0.47 | 0.11 | **0.29** | 1.56E-07 | 41 | 124 | 0.47 | 1.41 | **3.04** | 2.66E-07 |
|  | miR-146a | 7 | 1 | 0.08 | 0.01 | **0.17** | 2.39E-06 | 7 | 18 | 0.08 | 0.20 | **2.58** | 6.64E-05 |
|  | miR-1179 | 7 | 1 | 0.08 | 0.01 | **0.17** | 6.91E-06 | 7 | 17 | 0.08 | 0.19 | **2.44** | 1.27E-05 |
|  | novel_mir158 | 302 | 28 | 3.37 | 0.33 | **0.11** | 4.60E-08 | 302 | 1269 | 3.37 | 14.37 | **4.22** | 2.81E-08 |
|  | miR-378f | 15231 | 948 | 170.46 | 10.85 | **0.07** | 8.61E-12 | 15231 | 140459 | 170.46 | 1591.56 | **9.27** | 7.65E-12 |
|  | novel_mir204 | 10 | 2 | 0.11 | 0.01 | **0.06** | 2.17E-05 | 10 | 21 | 0.11 | 0.24 | **2.11** | 9.41E-05 |
|  | novel_mir326 | 10 | 2 | 0.12 | 0.01 | **0.06** | 4.96E-05 | 10 | 29 | 0.12 | 0.33 | **2.91** | 8.40E-05 |
|  | novel_mir319 | 11 | 1 | 0.12 | 0.01 | **0.05** | 1.39E-05 | 11 | 25 | 0.12 | 0.28 | **2.28** | 2.24E-06 |
|  | novel_mir31 | 11 | 1 | 0.13 | 0.01 | **0.05** | 6.07E-05 | 11 | 22 | 0.13 | 0.25 | **2.01** | 9.11E-05 |
|  | novel_mir110 | 11 | 2 | 0.13 | 0.01 | **0.05** | 5.52E+02 | 11 | 24 | 0.13 | 0.27 | **2.19** | 5.65E-05 |
|  | novel_mir17 | 13 | 1 | 0.15 | 0.01 | **0.05** | 1.19E-06 | 13 | 34 | 0.15 | 0.39 | **2.63** | 2.00E-07 |
|  | novel_mir387 | 16 | 1 | 0.19 | 0.01 | **0.04** | 4.12E-05 | 16 | 34 | 0.19 | 0.39 | **2.14** | 2.57E-07 |
