# Supplementary material for: A miR-125b/CSF1-CX3CL1/tumor-associated macrophage recruitment axis controls testicular germ cell tumor growth
Source: Cell Death Dis. 2018 Sep 20;9(10):962. doi: 10.1038/s41419-018-1021-z (PMC6148032; doi:10.1038/s41419-018-1021-z)
Supplement: Supplementary file 4 — supplementary figure legends [file 41419_2018_1021_MOESM4_ESM.docx]

**Fig. S1** **Efficiency of miR-125b knockdown and overexpression.** Relative miR-125b level in miR-125b agomir-, antagomir-, and NC-transfected NCCIT tumor cells was shown. *** indicated *p*<0.0001 by student’s *t* test. Data were presented as the mean±SEM (*n*=3 each).

**Fig. S2** **No effect of CSF1 and CX3CL1 on NCCIT cell proliferation/apoptosis *in vitro*. a** *CSF1* mRNA level was significantly downregulated by transfection of *CSF1* siRNAs. **b, c** Representative EdU incorporation staining and the percentage of EdU-positive cells were shown. **d, e** Representative TUNEL staining and quantification of fraction of TUNEL-positive cells were shown. **f** Relative *CX3CL1* mRNA level after gene silencing. **g, h** EdU incorporation assay. **i, j** TUNEL staining. ** indicated *p*<0.01 by student’s *t* test. Data were presented as the mean±SEM (*n*=3 each). Scale bar in B, D, G and I, 25 μm.

**Fig. S3 ELISA assay of CSF1 and CX3CL1.** Concentrations of CSF1 (pg/ml) and CX3CL1 (pg/ml) in miR-125b agomir-, antagomir-, and NC-treated NCCIT cells (green section) and their culture supernatant (white section) were detected by ELISA assay. * indicated *p*<0.05 and ** indicated *p*<0.01 by student’s *t* test. Data were presented as the mean±SEM (*n*=3 each).

**Table S1 RNA sequencing data** Differentially expressed genes among miR-125b agomir (ago)-, miR-125b antagomir (ant)-, and negative control (NC)-transfected NCCIT tumor cells by RNA sequencing were listed.

**Table S2 Cluster analysis data** Cluster analysis revealed the top enriched pathways under the regulation of miR-125b.

**Table S3 miRNA sequencing data** Differentially expressed miRNAs among miR-125b ago-, ant-, and NC-treated NCCIT tumor cells by miRNA sequencing were listed.
