# Supplementary figures and images for: A miR-125b/CSF1-CX3CL1/tumor-associated macrophage recruitment axis controls testicular germ cell tumor growth
Source: Cell Death Dis. 2018 Sep 20;9(10):962. doi: 10.1038/s41419-018-1021-z (PMC6148032; doi:10.1038/s41419-018-1021-z)

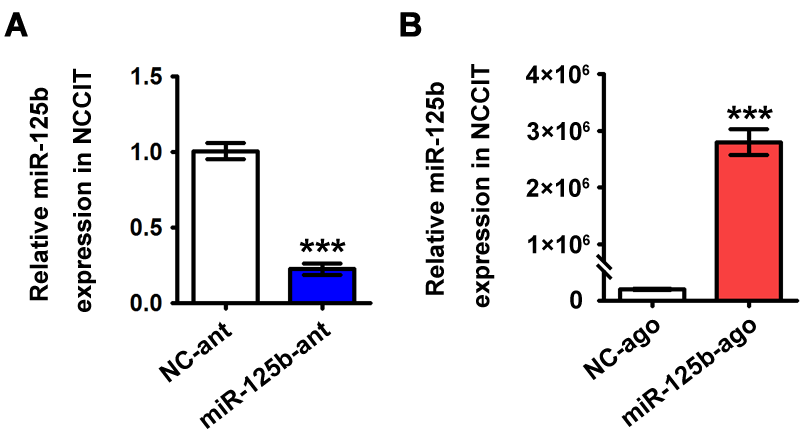

Supplement: Supplementary file 5 — Fig.S1 [file 41419_2018_1021_MOESM5_ESM.tif]

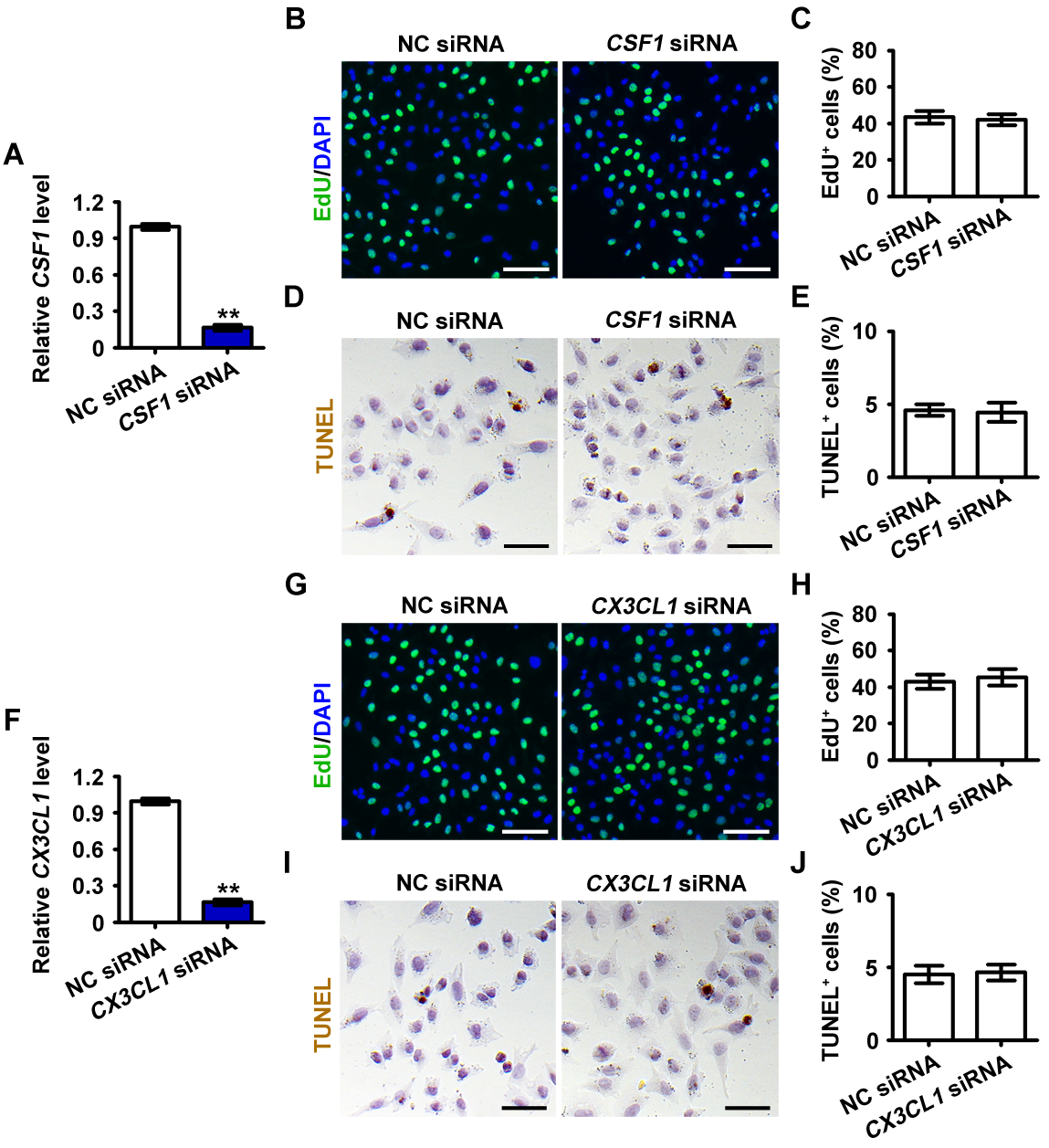

Supplement: Supplementary file 6 — Fig.S2 [file 41419_2018_1021_MOESM6_ESM.tif]

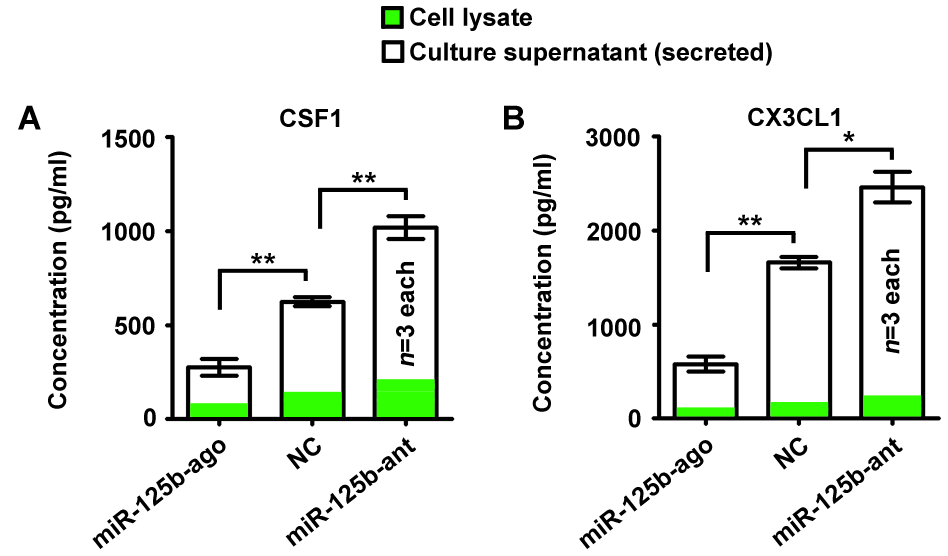

Supplement: Supplementary file 7 — Fig.S3 [file 41419_2018_1021_MOESM7_ESM.tif]
